# POCOP-Ni(II) pincer compounds derived from phloroglucinol. Cytotoxic and antioxidant evaluation

**DOI:** 10.3389/fchem.2024.1483999

**Published:** 2024-11-20

**Authors:** Andrés Amaya-Flórez, Juan S. Serrano-García, Jordi Ruiz-Galindo, Antonino Arenaza-Corona, J. Antonio Cruz-Navarro, Adrian L. Orjuela, Jorge Alí-Torres, Marcos Flores-Alamo, Patricia Cano-Sanchez, Viviana Reyes-Márquez, David Morales-Morales

**Affiliations:** ^1^ Instituto de Química, Universidad Nacional Autónoma de México, Ciudad de México, Mexico; ^2^ Departamento de Química, Universidad Nacional de Colombia-Sede Bogotá, Bogotá, Colombia; ^3^ Facultad de Química, División de Estudios de Posgrado, Universidad Nacional Autónoma de México, Mexico city, Mexico; ^4^ Departamento de Ciencias Químico-Biológicas, Universidad de Sonora, Hermosillo, Sonora, Mexico

**Keywords:** cytotoxic evaluation, antioxidant properties, pincer compounds, POCOP-Ni(II) complexes, metallopharmaceuticals

## Abstract

POCOP-Ni(II) pincer compounds have primarily been explored as catalysts, but their potential biological activity has been scarcely studied. To address this gap, we evaluated the anticancer and antioxidant potential of four POCOP-Ni(II) complexes derived from phloroglucinol. A comprehensive supramolecular analysis, based on single-crystal X-ray diffraction (DRX) structures, was conducted using Hirshfeld surfaces and non-covalent interaction analysis. The cytotoxicity of all complexes was systematically assessed against various cancerous cell lines, as well as a non-cancerous cell line (COS-7). The results revealed that complexes **1b** and **1c** exhibited remarkable antiproliferative activity, with IC_50_ values ranging from 2.43 to 7.85 μM against cancerous cell lines U251, K562, HCT-15, MCF-7, and SK-LU-1. To further elucidate their mechanism of action, a competitive fluorescence displacement assay with ethidium bromide (EB) suggested that these complexes possess the ability to intercalate with DNA. This multifaceted investigation not only enhances our understanding of the biological potential of POCOP-Ni complexes but also provides valuable insights into their structural features and interactions, paving the way for future exploration in both catalytic and therapeutic domains.

## Graphical Abstract

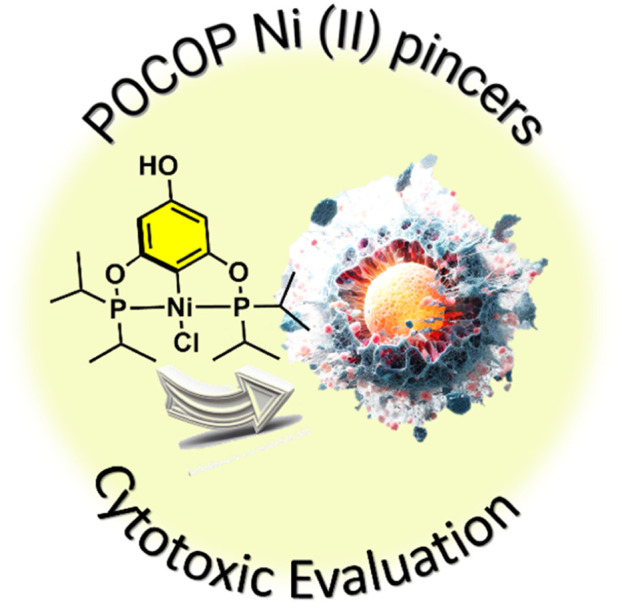

## 1 Introduction

Cancer, characterized by uncontrolled cell growth and the invasion of surrounding tissues, stands as one of the most formidable challenges in modern medicine (Marchi et al., 2022). The origins of cancer are often attributed to genetic mutations that disrupt normal cellular regulatory mechanisms, leading to aberrant signaling pathways and unchecked cell division (González-Ballesteros et al., 2022). This disease is broadly classified according to tissue origin and pathological characteristics. In this context, carcinomas arise from epithelial tissues, sarcomas from connective tissues, lymphomas from the lymphatic system, and leukemia from blood-forming tissues. Additionally, cancers are categorized by their degree of differentiation, histological features, and molecular signatures, contributing to a nuanced understanding of the disease landscape (Carbone, 2020).

In 2020, the World Health Organization (WHO) reported more than 19 million cases of cancer globally, of which 10 million resulted in death (Sung et al., 2021). This significant number of deaths has raised concerns about the future of cancer treatments. Since its discovery, cisplatin and its derivatives have remained a powerful tool in the treatment of several types of cancer; nonetheless, these compounds display severe side effects on the human body, including nephrotoxicity, ototoxicity (Wheate et al., 2010; Dilruba and Kalayda, 2016; Qi et al., 2019), and the development of resistance over time. These limitations have increased interest in exploring transition metals to develop novel and selective metallodrugs for cancer therapy.

Nickel, located in group 10 of the transition metals alongside platinum, plays a significant role in diverse biological systems, where it exerts its significance through incorporation into essential metalloproteins, such as urease (Maroney and Ciurli, 2014), [NiFe]-hydrogenase, (Ahmed and Dey, 2019), Acetyl-CoA synthase (Can et al., 2014), Ni-SOD (Wodrich and Hu, 2017), [NiFe]-CO dehydrogenase and lactate racemase (Xu et al., 2016). As a result, Ni(II) complexes have attracted considerable attention as potential anticancer agents due to their similarity to Pt (II) coordination geometry and DNA cross-linking properties (Hernández-Romero et al., 2021).

Although a wide variety of Ni(II) coordination and organometallic complexes with biological and anticancer activity have been extensively documented, the assessment of cytotoxic effects related to Ni(II) pincer complexes remains underexplored, with only a few papers addressing their antibacterial, (Soliman et al., 2019; Shukla et al., 2021), antimicrobial, and anticarcinogenic properties (Hosseini-Kharat et al., 2019; Kim et al., 2020; Muñoz-Patiño et al., 2020). On the other hand, pincer complexes have been increasingly synthesized due to their thermal stability and robustness, leading to various applications as catalysts, sensors, and dendrimers (Bedford et al., 2000; Morales-Morales et al., 2000a; 2000b; Morales-Morales, 2008; van Koten and Gossage, 2016). This growing interest in the multifaceted applications of pincer complexes highlights the need for a more comprehensive exploration of their cytotoxic potential. The limited information on the cytotoxic effects of these specific complexes underscores a significant gap in our knowledge, prompting further investigation to elucidate their potential as anticancer agents.

**FIGURE 1:**
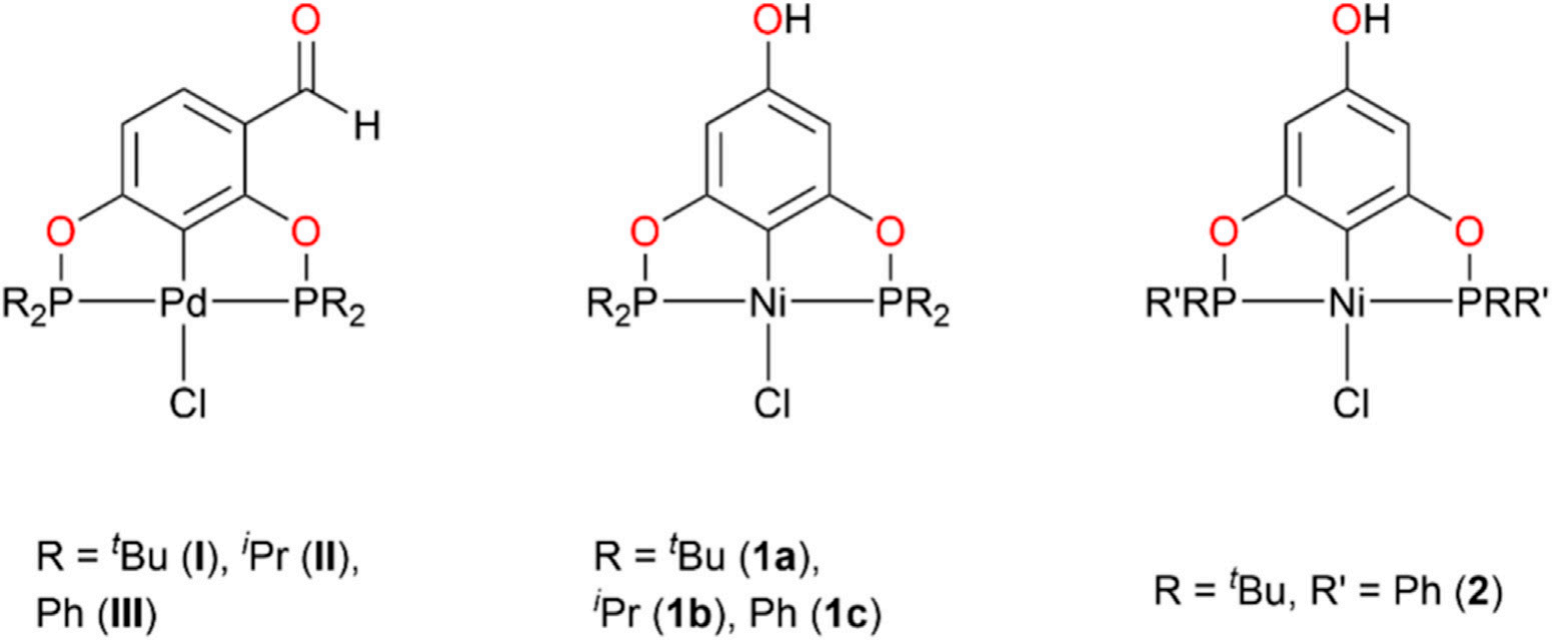
POCOP-Pd(II) pincer complexes previously used as antibacterial agents (**I-III**), and POCOP-Ni(II) pincer complexes evaluated in this work as antitumoral and antioxidant agents (**1a-c**, **2**).

In the context of our previous research, we reported the synthesis and antibacterial activity of an important series of POCOP-Pd(II) pincer complexes (**I-III**, Figure 1). These complexes exhibited notable antibacterial efficacy, with MIC values approximately 8 μg mL^−1^ against the *S. aureus* ATCC 25923 strain (Aragón-Muriel et al., 2022). Furthermore, molecular docking studies revealed significant interactions with KPC-2 and PBP2A enzymes, providing valuable mechanistic insights.

Building on previous investigations, our research team has also explored the synthesis of POCOP-Ni(II) pincer compounds. However, despite these earlier studies, the cytotoxic activity of these Ni(II) counterparts remains unexplored. In this study, we focused on the biological activity of three *para*-hydroxy POCOP-Ni(II) pincer complexes (**1a-c**) previously reported (García-Eleno et al., 2015), and a novel compound (2) newly synthesized. All complexes were tested against six cancer cell lines (U251, PC-3, K562, HCT-15, MCF-7, SK-LU-1) and a non-cancerous line (COS-7). The findings from this work enhance our understanding of the potential applications of POCOP pincer complexes in metallodrug development.

## 2 Results and discussion

**SCHEME 1:**
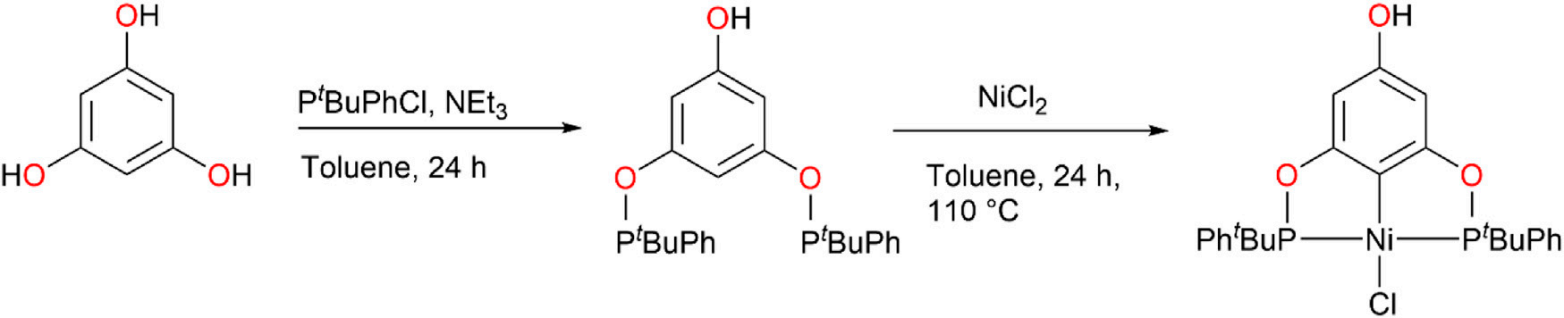
Synthesis of **2**.

The pincers **1a-c** (R = ^*t*^Bu, ^*i*^Pr, Ph, respectively) were synthesized according to the methodology previously reported by our investigation group and their spectroscopic characterization correlated perfectly (García-Eleno et al., 2015). In addition, compound **2** (where R = ^*t*^Bu and R’ = Ph) was synthesized from phloroglucinol, chloro (*tert*-butyl)phenylphosphine, and anhydrous NiCl_2_ (see Scheme 1 and details in the experimental part). All compounds were characterized by ^1^H, ^13^C{^1^H}, and ^31^P{^1^H} NMR, DART^+^ MS, ATR-FTIR, and elemental analysis, obtaining the expected signals (see Supplementary Figures S1–S5). Regarding ^31^P{^1^H} NMR, the spectroscopic characterization showed signals at 189.8 ppm (**1a**), 187.3 (**1b**) 147.7 (**1c**). For complex **2**, two ^31^P NMR signals were obtained at 160.5 and 159.7 ppm, which would correspond to the racemic and meso isomers (See Supplementary Figure S3). As shown in ^1^H NMR spectrum, these isomers are present in a 1:1 ratio. Interconversion between the meso and racemic isomers have not been observed during analysis and have not been reported for similar POCOP-Ni(II) (Adhikary et al., 2015).

**FIGURE 2:**
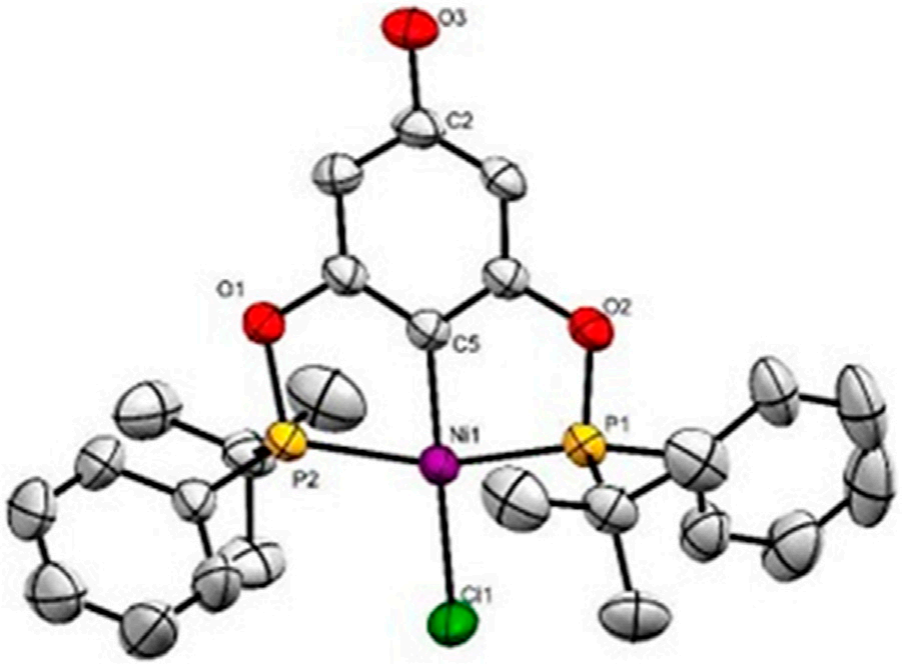
Molecular structure of **2-*rac***. The ellipsoids are shown at 50% of probability level and hydrogen atoms have been omitted for clarity. Selected bonds (Å) distances and angles (°) for **2-*rac***: Ni(1)-Cl (1) 2.2236 (9), Ni(1)-C (5) 1.892 (3), Ni(1)-P (1) 2.1824 (9), Ni(1)-P (2) 2.1808 (9), P (2)-Ni(1)-P (1) 162.43 (4), C (2)-Ni(1)-Cl (1) 179.6 (1), P (2)-C (5)-P (1) 108.0 (1).

Crystallization of complex **2** from 1:1 CH_2_Cl_2_/MeOH resulted in the crystallization of the racemic isomer (**2-*rac*).** Its structure was determined by single-crystal X-ray diffraction (XRD) and molecular structure is shown in Figure 2; crystal data and other information are presented in Supplementary Table S1. Complex **2-*rac*** crystallized in an orthorhombic system (*Pbca*), and as seen for the analogous structures (García-Eleno et al., 2015) the metal atom is tri-coordinated by the POCOP pincer and the fourth position is occupied by a chlorine atom adopting a square planar geometry, the heteroalquil (RR’P-Ni-PR’R) substituents on the phosphine ligands prefers to adopt the *anti-*disposition. The crystal arrangement is mainly stabilized by a hydrogen bond along the *a*-axis between the chloride ligand and the hydrogen of the *para*-hydroxyl group, with a Cl (1)-O (3) distance of 3.281 Å [-1/2 + x, y, 1/2-z].

**FIGURE 3:**
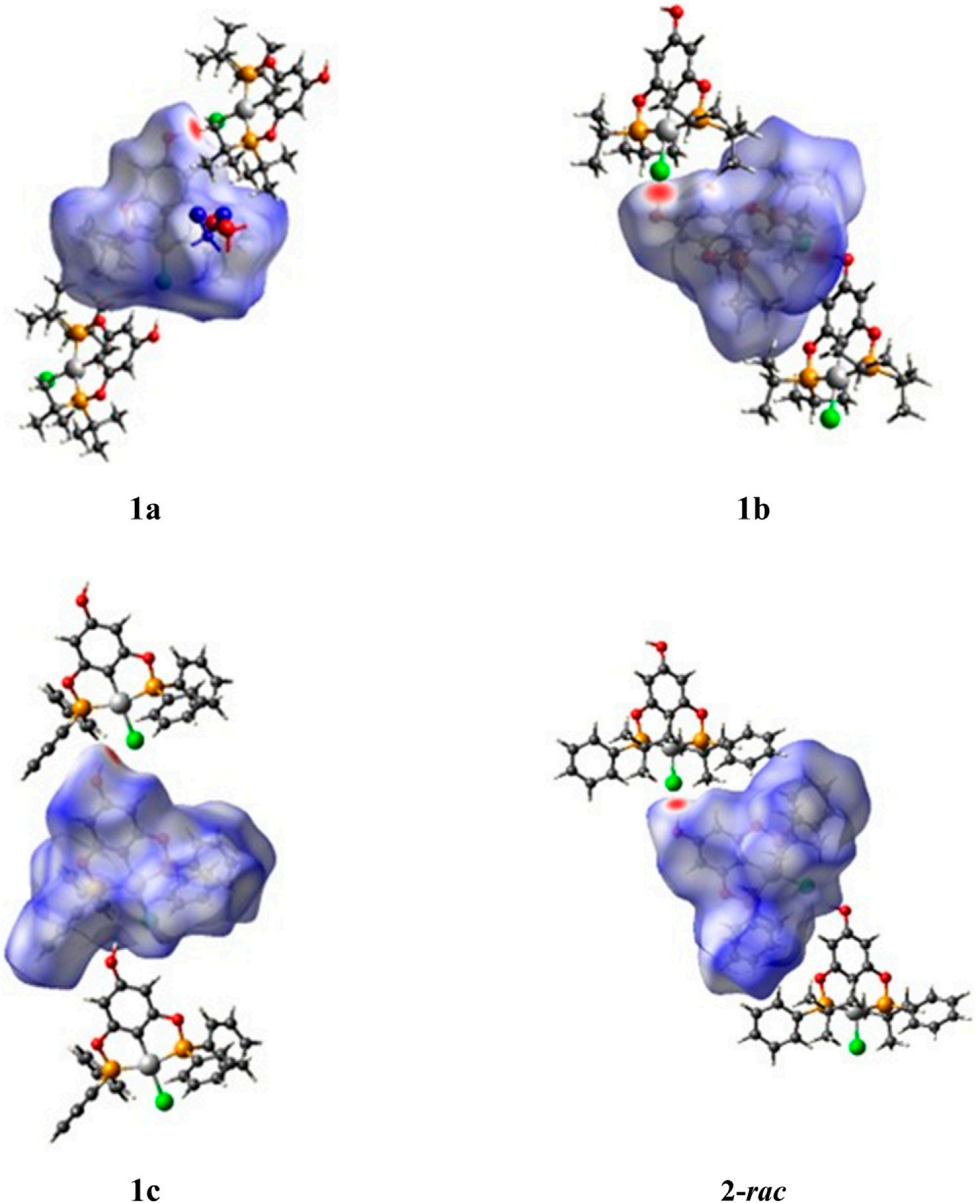
Hirshfeld surfaces calculated for complexes **1a**, **1b**, **1c**, and **2-*rac***. Note: except compound **2-*rac***, CIF data were taken from García-Eleno et al. (2015) and only one of both molecules of the asymmetric unit of **1b** was plotted.

**CHART 1:**
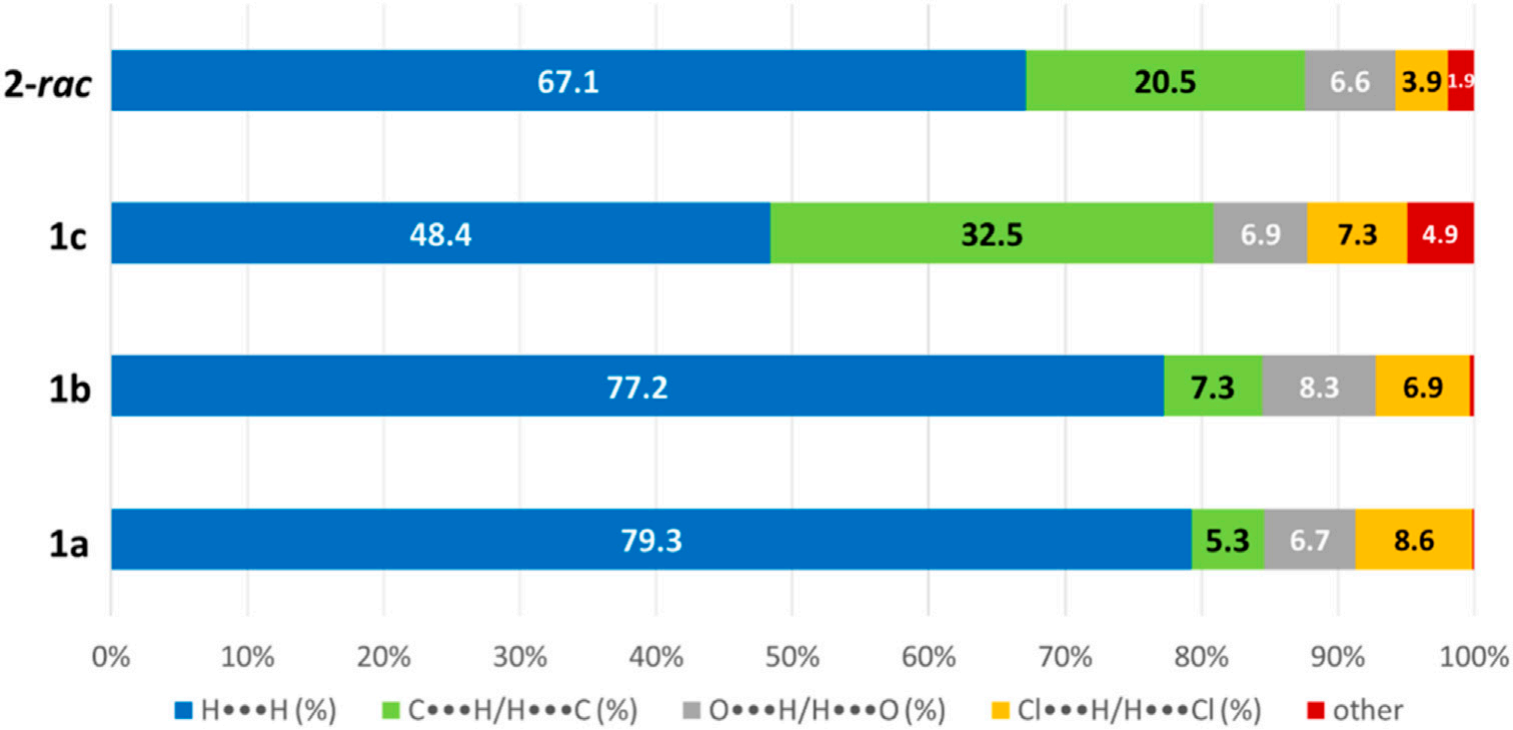
Plot of percentages of contacts observed in complexes **1a**, **1b**, **1c**, and **2-*rac***.

To obtain further information on the nature of the molecular packing, the Hirshfeld surface analysis was drawn by using *CrystalExplorer* software using the CIF file from X-ray studies (Spackman et al., 2021). The surface of compound **2-*rac*** was mapped over the d_norm_ function, along with the congeneric compounds (García-Eleno et al., 2015) for comparative reasons, which are illustrated in Figure 3. The red regions demonstrate close contacts (shorter than the sum of van der Waals radii) and are located in the *para*-hydroxyl group in all the complexes, resulting from contact with chlorine atom (OH···Cl) with distances within the ranges of d (D-A) = 3.127–3.281 Å supporting 1D chains at the supramolecular level, the graph set descriptor found for the four structures was C (8) due to they contain the same skeleton (see representation in capped stick in Supplementary Figure S6 in the Supplementary Material). Additionally, 2D Fingerprint (Spackman and McKinnon, 2002) plots were generated and are listed in Table 1. Apparently, the fingerprints are very similar to each other, reflecting the similar types of interactions found in the crystalline arrangement. However, the percentages of contributions vary considerably; the majority of contributions were H···H, O···H/H···O and Cl···H/H···Cl contacts. The last one was identified as two characteristic symmetrical spikes. Individual contributions are summarized in Chart 1.

**TABLE 1:** Representative fingerprints of non-covalent interactions of **1a**, **1b, 1c**, and **2-*rac***. Distances ***d***_***e***_ (*y*-axis) and ***d***_***i***_ (*x*-axis) are in Å.

Compound	Cl•••H/H•••Cl (%)	O•••H/H•••O (%)	C•••H/H•••C (%)	H•••H (%)
**1a**	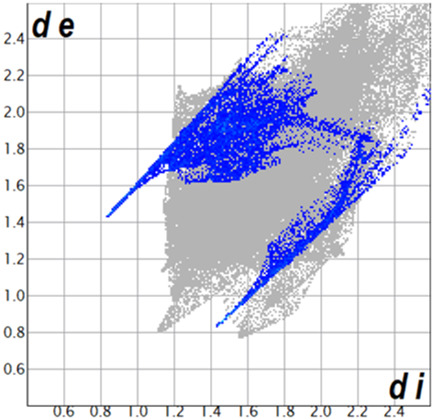 8.6	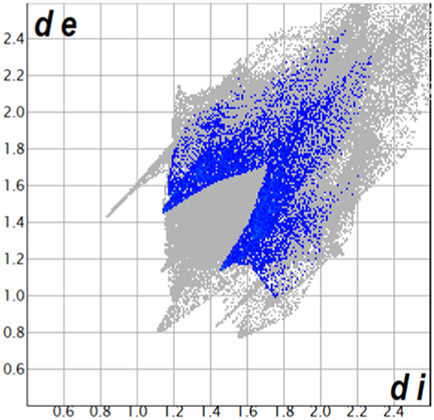 6.7	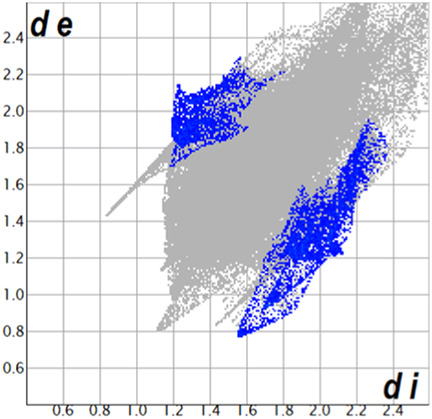 5.3	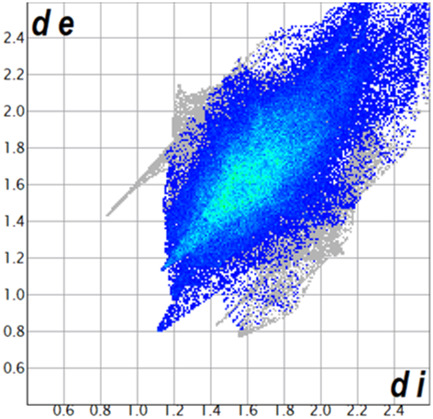 79.3
**1b**	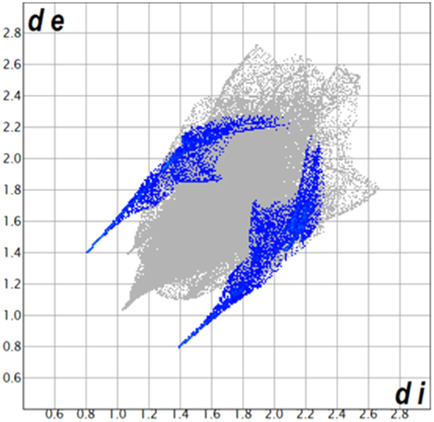 6.9	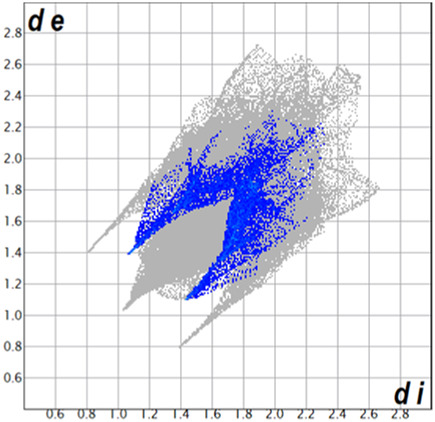 8.3	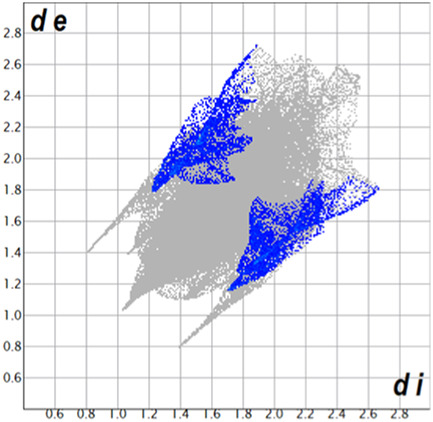 7.3	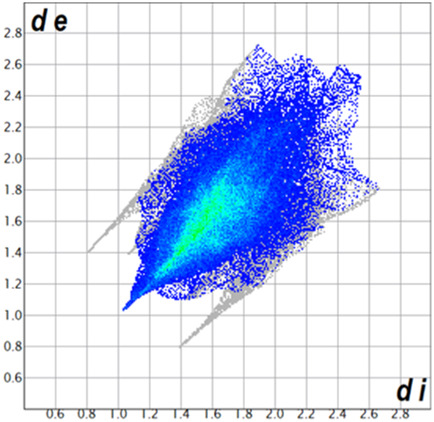 77.2
**1c**	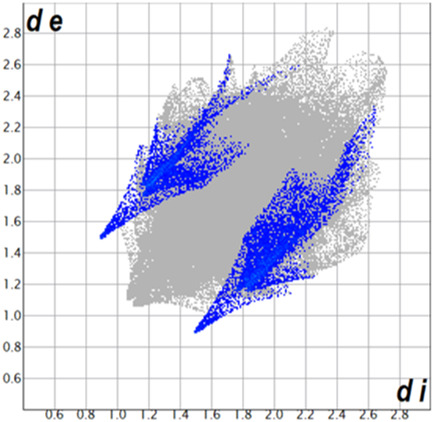 7.3	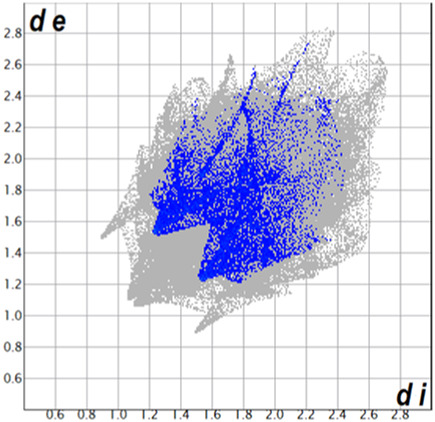 6.9	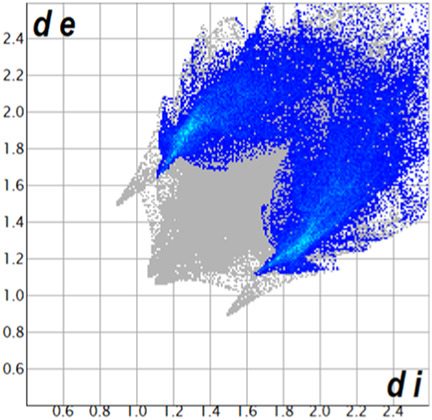 32.5	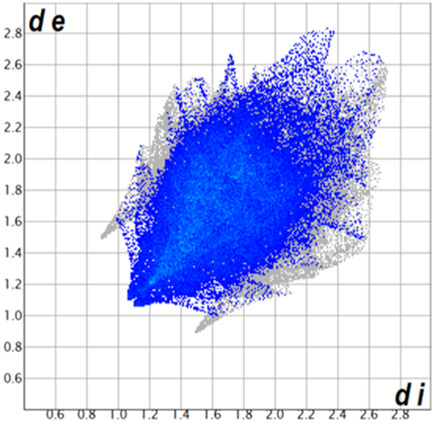 48.4
**2-*rac***	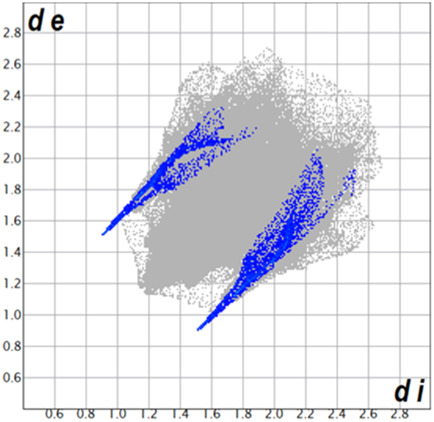 3.9	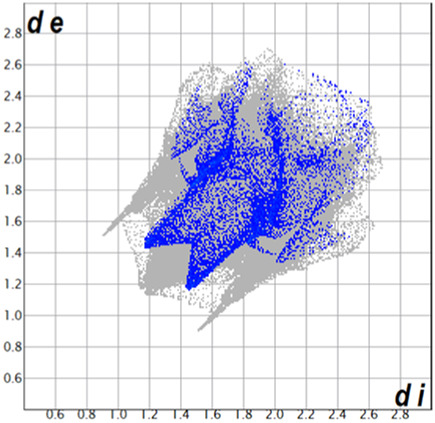 6.6	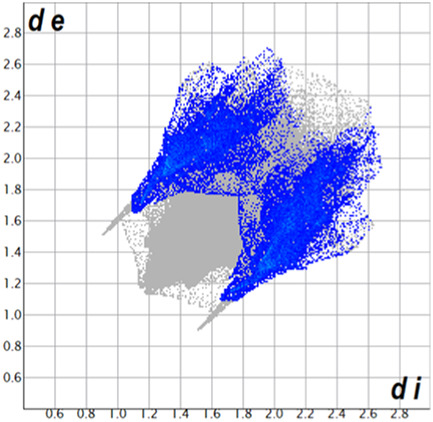 20.5	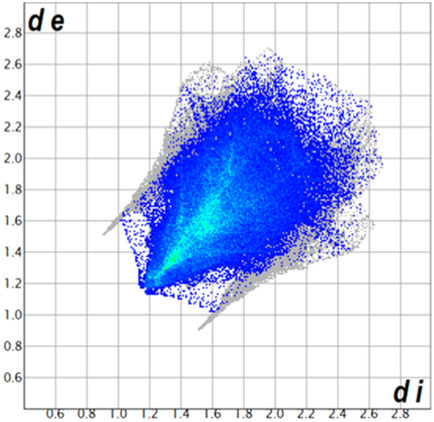 67.1

Quantitative and qualitative structure-activity relationships can be established between noncovalent interactions from Hirshfeld surface analysis of biologically active compounds and their biological activity (Małecka and Budzisz, 2014; Kupcewicz et al., 2016; Małecka et al., 2020). In our study, a notorious variation is observed in the percentage contribution of C•••H/H•••C contacts, with a maximum value of 32.5% for **1c** and a minimum value of 5.3% for **1a.** Interestingly, this trend is also observed with their cytotoxicity and their calculated interactions with DNA (*vide infra*).

## 3 Cytotoxic activity of POCOP-Ni(II) pincer complexes

Although complexes **1a**, **1b**, and **1c** have previously been reported by our research group (García-Eleno et al., 2015), no studies related to their cytotoxic activity have been made. Therefore, a preliminary evaluation of the *in vitro* cytotoxic activity (Table 2) of these complexes, along with complex **2**, was carried out. The assays were performed using the sulforhodamine B protocol, with a concentration of 10 μM of the corresponding complex employing DMSO as a vehicle. Six human cancer cell lines were used: U251 (human glioblastoma), PC-3 (human prostate adenocarcinoma), K562 (human chronic myelogenous leukaemia), HCT-15 (human colorectal adenocarcinoma), MCF-7 (human breast adenocarcinoma) (these cell lines were provided by the National Cancer Institute, United States of America), SK-LU-1 (human lung adenocarcinoma) (this cell line was donated by the Cancer Institute of Mexico). A healthy monkey kidney cell line (COS-7) was also included for comparative purposes. Based on the results obtained, complex **1b** exhibited the highest cytotoxic activity, showing a 100% inhibition rate against all cell lines, including COS-7, whereas complex **2** presented significant activity, except for K562. In contrast, complexes **1a** and **1c** were less toxic against COS7, with inhibition percentages of 47.5% and 79.3%, respectively. The difference in the biological activities of these compounds may be attributed to steric factors of the alkyl groups located on the phosphorus atoms, which could facilitate the release of chloride ions from the coordination sphere, creating a vacant space around the metal atom, allowing it to interact with specific biological targets. Additionally, solubility may facilitate the optimal transport of these compounds. However, further experimental studies are needed to validate these hypotheses.

**TABLE 2:** Growth inhibition (100%) of cancer cell lines by pincer complexes (10 μM).

Compound	U251	PC-3	K562	HCT-15	MCF-7	SK-LU-1	COS7
**1a**	44.3	61.2	48.3	55.7	63.6	98.1	47.5
**1b**	100	100	100	100	100	100	100
**1c**	100	94.1	81.7	81.5	26.8	100	79.3
**2**	100	92.9	NC	100	98.7	100	100

Moreover, the IC_50_ values for complexes **1b** and **1c** were determined across five cancer cell lines (U251, K562, HCT-15, MCF-7, and SK-LU-1), with *cisplatin* serving as the control drug (Table 3). Notably, complex **1c** demonstrated superior activity compared to complex **1b** in multiple cancer cell lines, except for the HCT-15 cell line (7.71 ± 0.6 μM), where complex **1b** exhibited greater efficacy (6.84 ± 0.9 μM).

**TABLE 3:** IC_50_ values for complexes **1b** and **1c** (μM).

Compound	U251	K562	HCT-15	MCF-7	SK-LU-1
**1b**	6.85 ± 0.08	5.81 ± 0.2	6.84 ± 0.9	7.85 ± 0.6	4.41 ± 0.04
**1c**	2.43 ± 0.3	3.85 ± 0.2	7.71 ± 0.6	6.98 ± 0.4	2.59 ± 0.2
***Cisplatin***	9.5 ± 0.7	1.2 ± 0.08	13.5 ± 0.7	17.9 ± 3.5	3.4 ± 0.5

In contrast, *cisplatin* exhibited superior activity against K562 (1.2 ± 0.08 μM) compared with complex **1b** and **1c**, and SK-LU-1 (3.4 ± 0.5 μM) in the case of complex **1b**.

## 4 Antioxidant activity of POCOP-Ni(II) pincer complexes

Antioxidant compounds have played a crucial role in the biological field, since they are believed to help prevent numerous diseases caused by the production of reactive oxygen species (ROS). Among these diseases, cancer is one that can be originated from the formation of free radicals and an overproduction of ROS. For that reason, the antioxidant activity of the pincer complexes was tested using the thiobarbituric acid reactive substances (TBARS) assay, which involves the production of ROS with FeSO_4_ in the presence of lipids obtained from rat brains. The assays were conducted using three different concentrations of the complexes under evaluation (1, 10, and 100 μM) (Table 4). It was observed that among the three complexes, compound **1a** demonstrated superior antioxidant activity, with an inhibition percentage of 94.77% at a concentration of 10 μM, compared to complexes **1b** and **1c**, which exhibited inhibition percentages at the same concentration of 40.45% and 32.86%, respectively. However, at a concentration of 100 μM, all complexes showed a high inhibition rate (>90%), indicating that the antioxidant activity of the complexes is concentration dependent. Similar to the cytotoxicity assays, a clear difference in biological activity can be observed when varying the alkyl groups on the phosphine moieties, which could be associated to the electronic effects. Thus, the *tert*-butyl groups would donate higher electron density to the metal and then to the aromatic ring, stabilizing the phenolate and reducing the lipid radical at a lower concentration (Marchi et al., 2022)

**TABLE 4:** Antioxidant activity of POCOP-Ni(II) pincer complexes through inhibition of lipid peroxidation (rat brain tissues).

Compound	Concentration (μM)	Inhibition (%)
**1a**	1 10 100	29.13 94.77 94.92
**1b**	1 10 100	24.35 40.45 94.62
**1c**	1 10 100	4.66 32.86 90.67
**2**	1 10 100	20.94 96.24 96.76

Homogenized in: PBS; vehicle: DMSO; peroxidation: induced with FeSO_4_ at 10 μM, 1 h of incubation; EDTA: 2 μM.

IC_50_ values of the complexes were determined by using butylhydroxytoluene (BHT) and α-tocopherol as controls (Table 5; Supplementary Table S2). Complexes **1a** and **1c** exhibited lower antioxidant activity compared to BHT and α-tocopherol (13.30 ± 0.77 μM and 19.29 ± 3.04 μM). On the other hand, complex **1b** displayed the best antioxidant activity among the three complexes, surpassing α-tocopherol and slightly less active than BHT (1.55 ± 0.08 μM). These results clearly show the effect of alkyl groups on the phosphine groups regarding their antioxidant activity.

**TABLE 5:** IC_50_ values (μM) for the antioxidant activity of POCOP-Ni(II) pincer complexes.

Compound	IC_50_ (μM)
**1a**	1.55 ± 0.08
**1b**	13.30 ± 0.77
**1c**	19.29 ± 3.04
**2**	2.19 ± 0.05
**BHT**	1.22 ± 0.44
**α-tocopherol**	2.16

## 5 Ethidium bromide displacement assay

**FIGURE 4:**
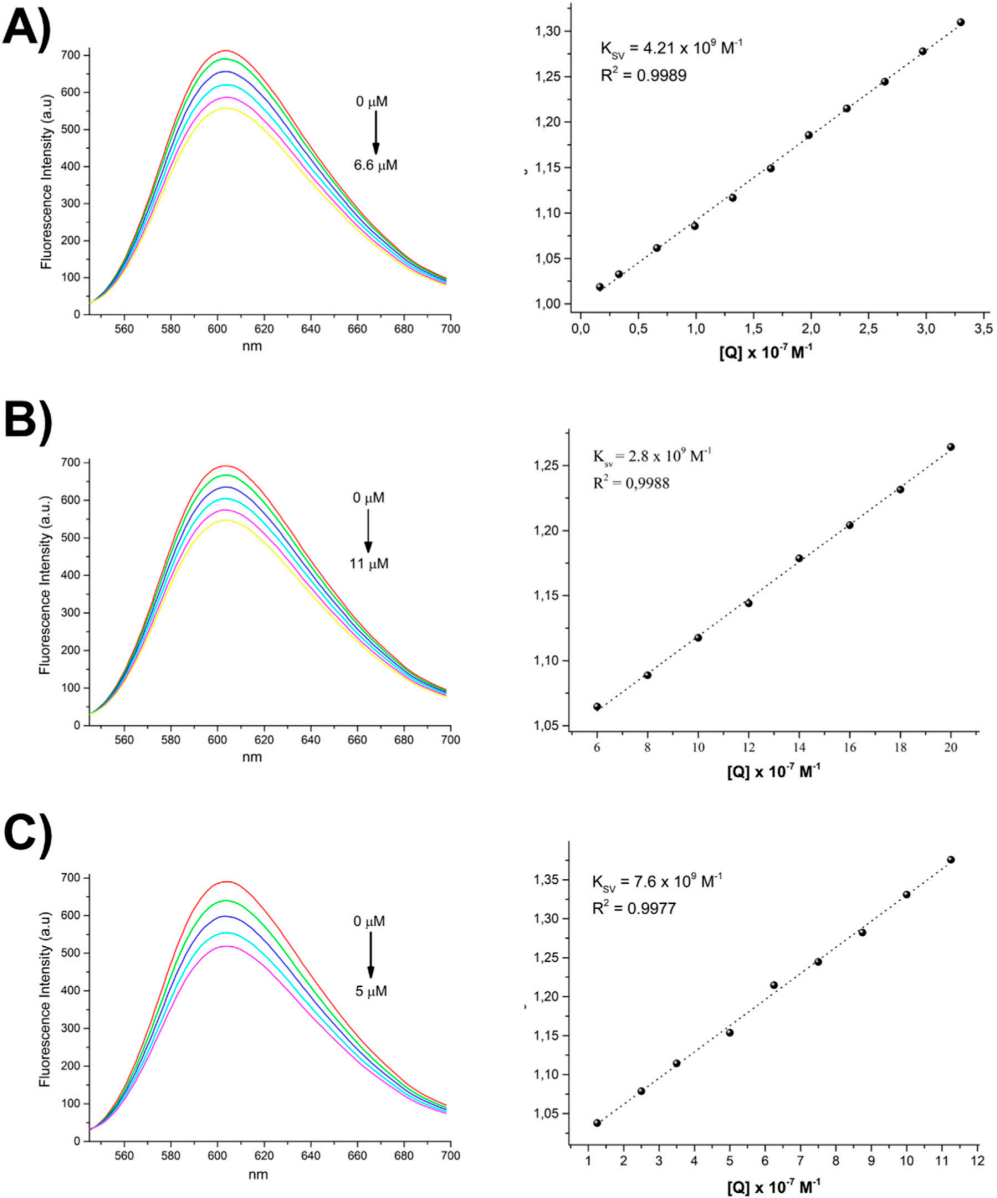
Fluorescence spectrum of EB-DNA in the presence of an increasing concentration of the **1a (A)**, **1b (B)** and **1c (C)** compounds. The arrows indicate changes in emission intensity as a function of complex concentration. On the right, Stern–Volmer plots of the fluorescence titration data are shown.

**FIGURE 5:**
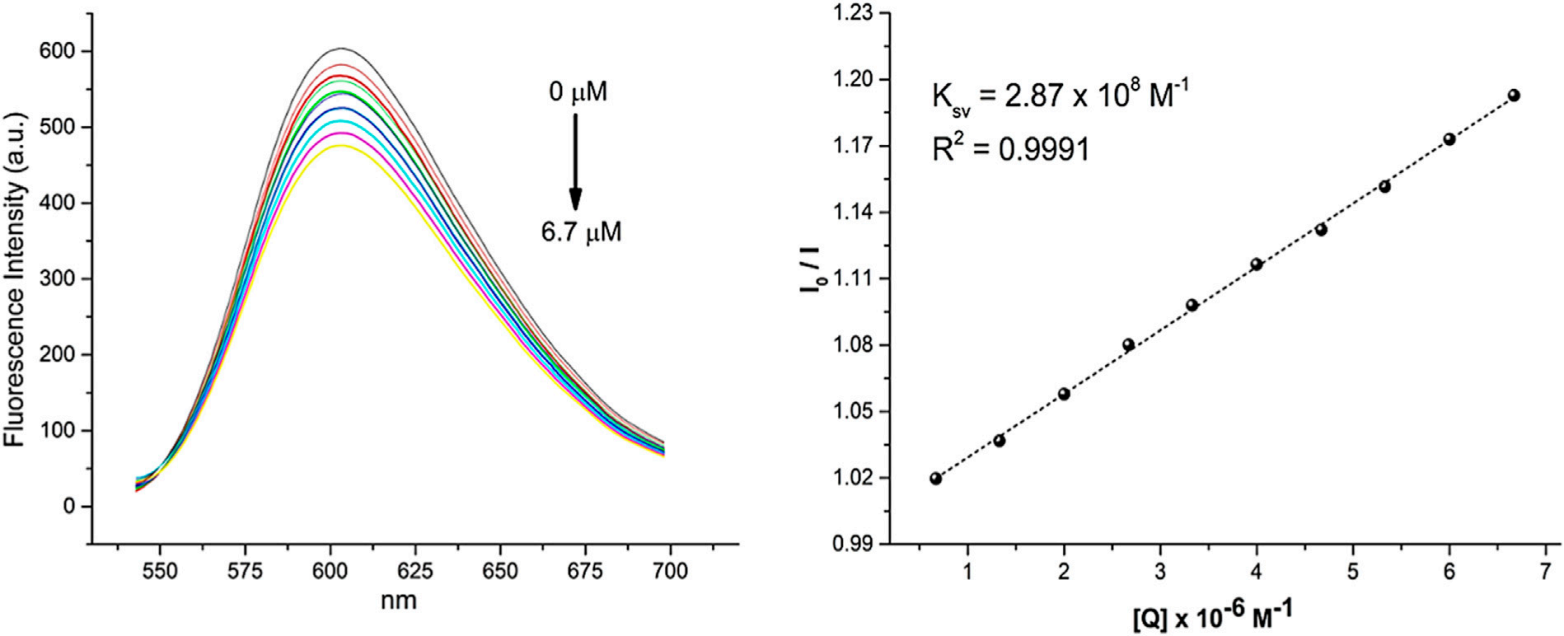
Fluorescence spectrum of EB-DNA in the presence of an increasing concentration of the **2**. The arrows indicate changes in emission intensity as a function of complex concentration. On the right, Stern–Volmer plots of the fluorescence titration data are shown.

To understand the interaction of **1a**, **1b**, **1c** and **2** complexes with DNA, competitive ethidium bromide (EB) fluorescence titration assays were performed (Figures 4, 5). EB is known to be a proficient intercalator and exhibits very weak fluorescence on its own. However, when intercalates with DNA the fluorescence increases considerably. If a compound has the ability to intercalate with DNA in the same manner as EB, a competition for the binding sites occurs, resulting in the release of EB from DNA. As a result, there is a modification in the fluorescence intensity of the EB-DNA adduct as the competing compound concentration in the system increases. As shown in Figures 4, 5, as the concentration of the **1a**, **1b**, **1c** and **2** complexes gradually increases from 0 to 6.7 μM, 0–11.0 μM, 0–5.0 μM and 0–6.7 μM, respectively, the fluorescence intensity of the EB-DNA adduct is progressively reduced. This behaviour indicates that the complexes intercalate between the DNA double helix, competing with EB for binding sites on the DNA and displacing EB, resulting in a decrease in fluorescence intensity. The K_SV_ value for the quenching of fluorescence intensity of EB bound to DNA by compounds **1a**, **1b**, **1c** and **2** was calculated from the Stern–Volmer plot, which showed good linearity in all cases, suggesting that the spectroscopic measurements are consistent with an intercalative mode of interaction between the pincer complexes and DNA. Additionally, it can be observed that the trend in the K_SV_ values increases in the order of **1c** > **1a** > **1b > 2**, with compound **1c** having the highest intercalative binding, possibly due to the presence of aromatic rings located over the phosphorus atoms.

## 6 Computational results

Based on the experimental results obtained, it was decided to carry out an *in silico* study to observe if there is a correlation between the experimental and theoretical parts, offering a valuable insight into the elucidation of the mechanism of action at the molecular level. Moreover, in this section, we will comprehensively analyze the binding interactions between the selected compounds and their molecular targets: DNA, topoisomerase I, and topoisomerase II. These targets were chosen because, on one hand, DNA is involved in cell replication, and inhibiting such replication in cancer cells leads to programmed cell death. Additionally, many drugs are targeted towards this objective. Regarding topoisomerases, I and II, inhibiting these enzymes delays the ligation stage of the cell cycle, which affects the cleavage of the DNA double helix. Molecular docking simulations, allow us to examine the binding sites, binding modes, and key molecular interactions, including hydrogen bonds, π-σ interactions, and hydrophobic interactions Therefore, molecular docking simulations of complexes **1a-1c** and **2** were performed on these biomacromolecules. For complex **2**, electronic structure was optimized from the racemic isomer (**2-*rac***), whose molecular structure was obtained by XRD.

### 6.1 Electronic structure and molecular docking protocol validation

To validate the electronic structure method, we utilized crystallized ligands as benchmarks and contrasted them with results obtained from electronic structure optimizations. Our analysis revealed Root Mean Square Deviation (RMSD) variances of less than 0.3 Å, primarily due to the limited flexibility of the complexes, as demonstrated in Supplementary Table S3.

**FIGURE 6:**
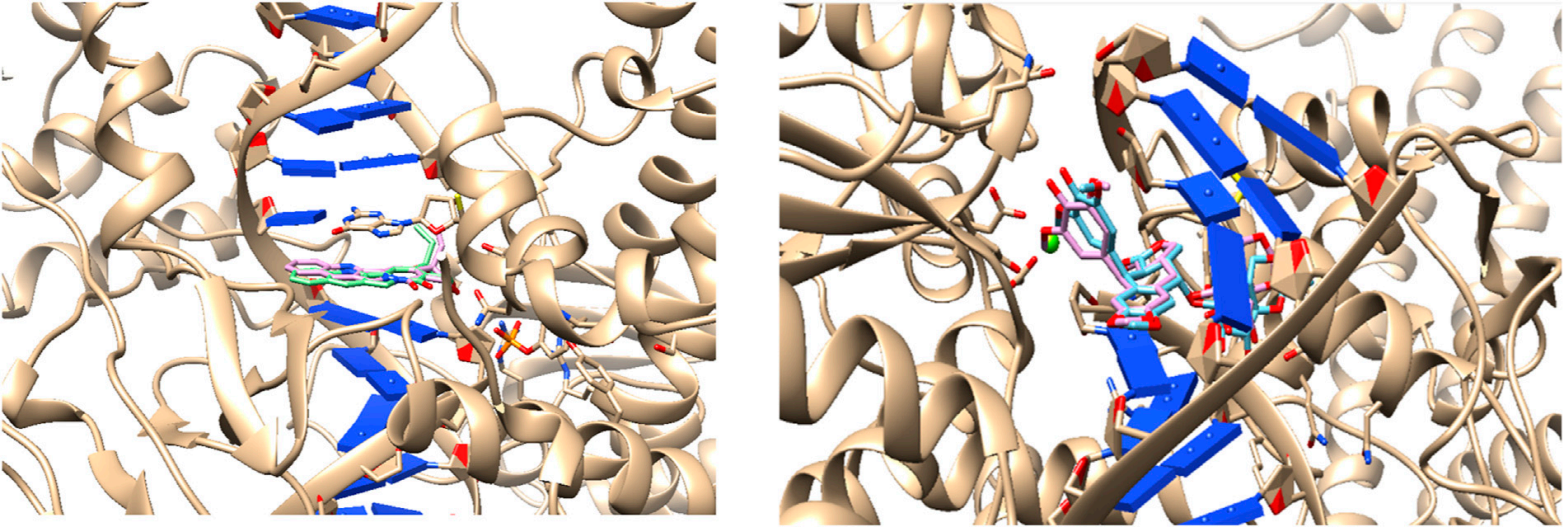
Validation of the molecular docking process. Left: Redocking of Topoisomerase I with Camptothecin as inhibitor. Right: Topoisomerase II with Etoposide as inhibitor.

The validation of the molecular docking method utilized crystallized ligands from structures available in the Protein Data Bank (PDB). Across all three programs, we observed RMSD values of less than 1.0 Å from the docking site, indicating the robustness of these programs in identifying docking sites within the three models of interest: DNA, Topoisomerase I, and Topoisomerase II. By employing multiple computational tools, we aim to cross-validate our results and identify the differences in interaction patterns across the selected targets. Additionally, we obtained affinity energies, which will serve as reference points for evaluating crystallized compounds. This outcome underscores the efficacy of our chosen computational approach in accurately modeling interactions within biologically relevant complexes, as depicted in Figure 6.

### 6.2 Molecular docking simulations

The analysis of the molecular docking simulations involving DNA, reveals that complexes **1b** and **1c** exhibited higher activity compared to cisplatin, which was used as a reference. These results agree with those obtained from experiments involving cellular lines (Table 6).

**TABLE 6:** Exponential Consensus Ranking (ERC) between DNA and the nickel complex.

Molecular docking program	1a	1b	1c	2-*rac*	*cisplatin*
Vina (kcal/mol)	−5.9	−6.8	−7.6	−7.0	−6.0
Smina (kcal/mol)	−5.6	−6.7	−7.1	−6.3	−5.7
ATD (kcal/mol)	−1.8	−1.9	−3.8	−2.4	−1.7
ERC	2.8	5.7	16.0	6.2	2.9

**FIGURE 7:**
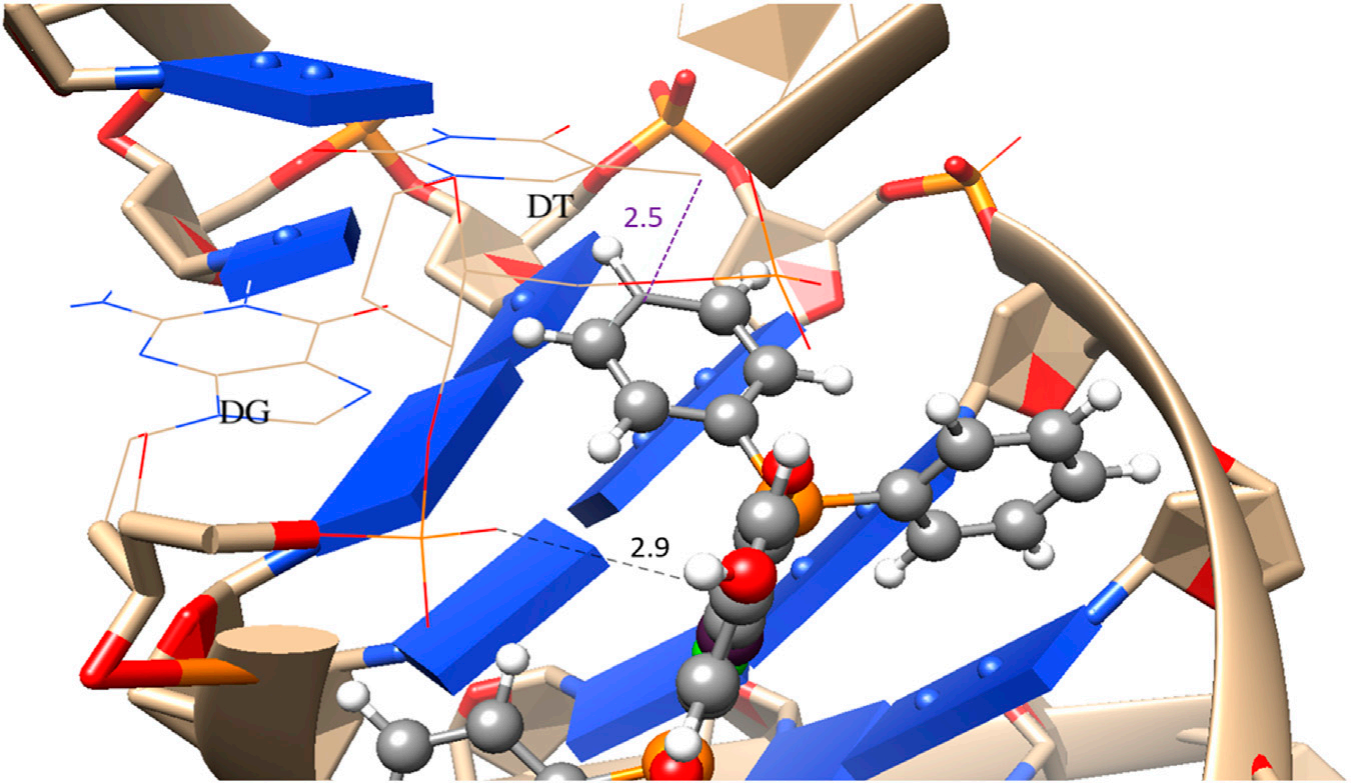
Coordination mode of DNA model with complex **1c**. Purple π-σ interaction, black π-anion. Distances are in Angstrom.

**FIGURE 8:**
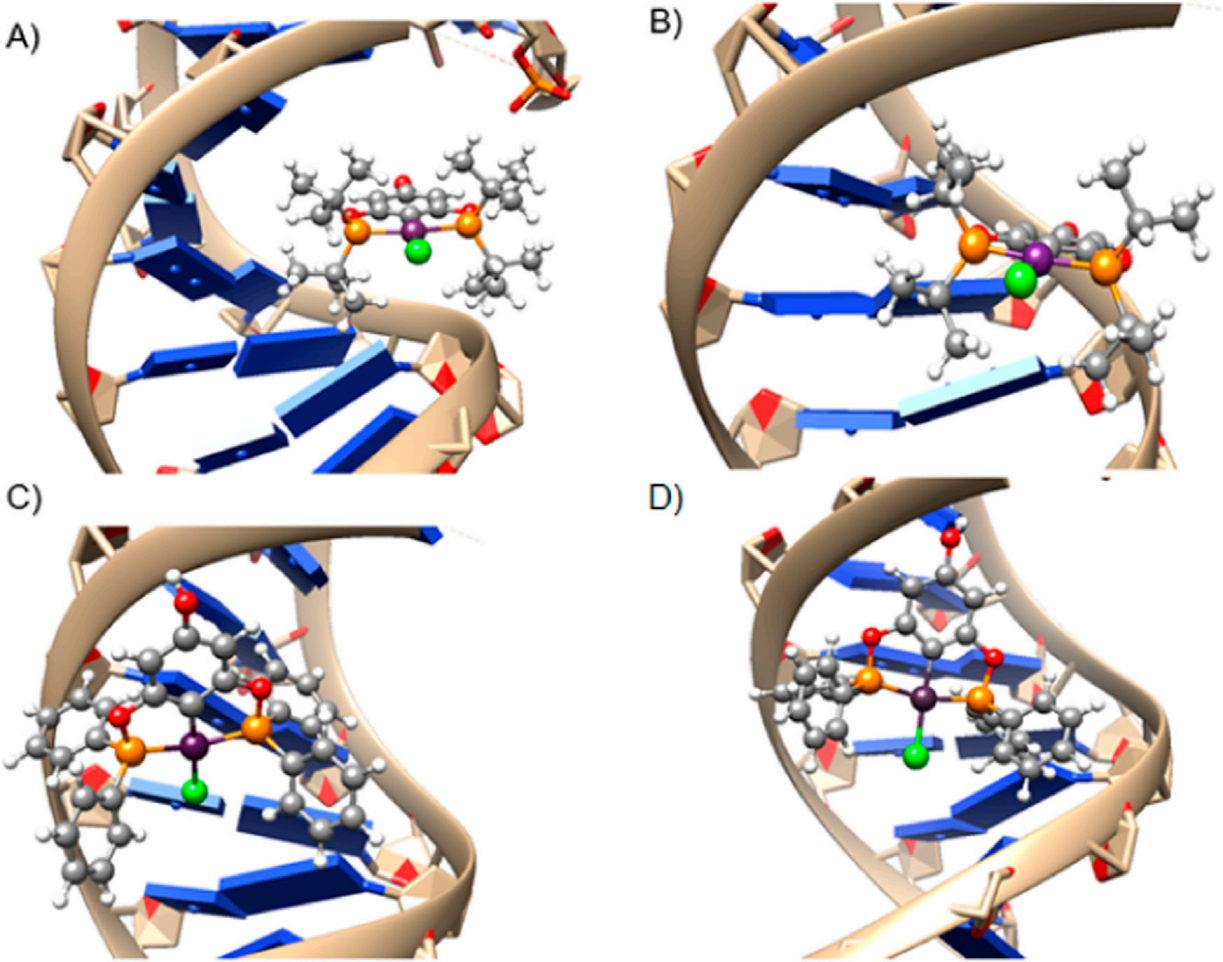
Representation of the most stable poses depicting the interaction between the DNA model and **(A) 1a**, **(B) 1b**, **(C) 1c**, and **(D) 2-*rac*** complexes.

In the molecular docking process, the primary interactions identified are π-σ interactions with phenolic rings (Figure 7). The heightened interactions observed with compounds **1c** and **2-*rac***, in contrast to the diminished interactions with compounds **1a** and **1b**, can be attributed to the latter inducing electrostatic repulsions with the tert-butyl groups in the case of molecule **1a** and isopropyl groups in molecule **1b** (Figure 8). This underscores a specificity in how these complexes interact with DNA, where geometric and electronic compatibility play crucial roles in determining their binding efficiency and biological activity.

**FIGURE 9:**
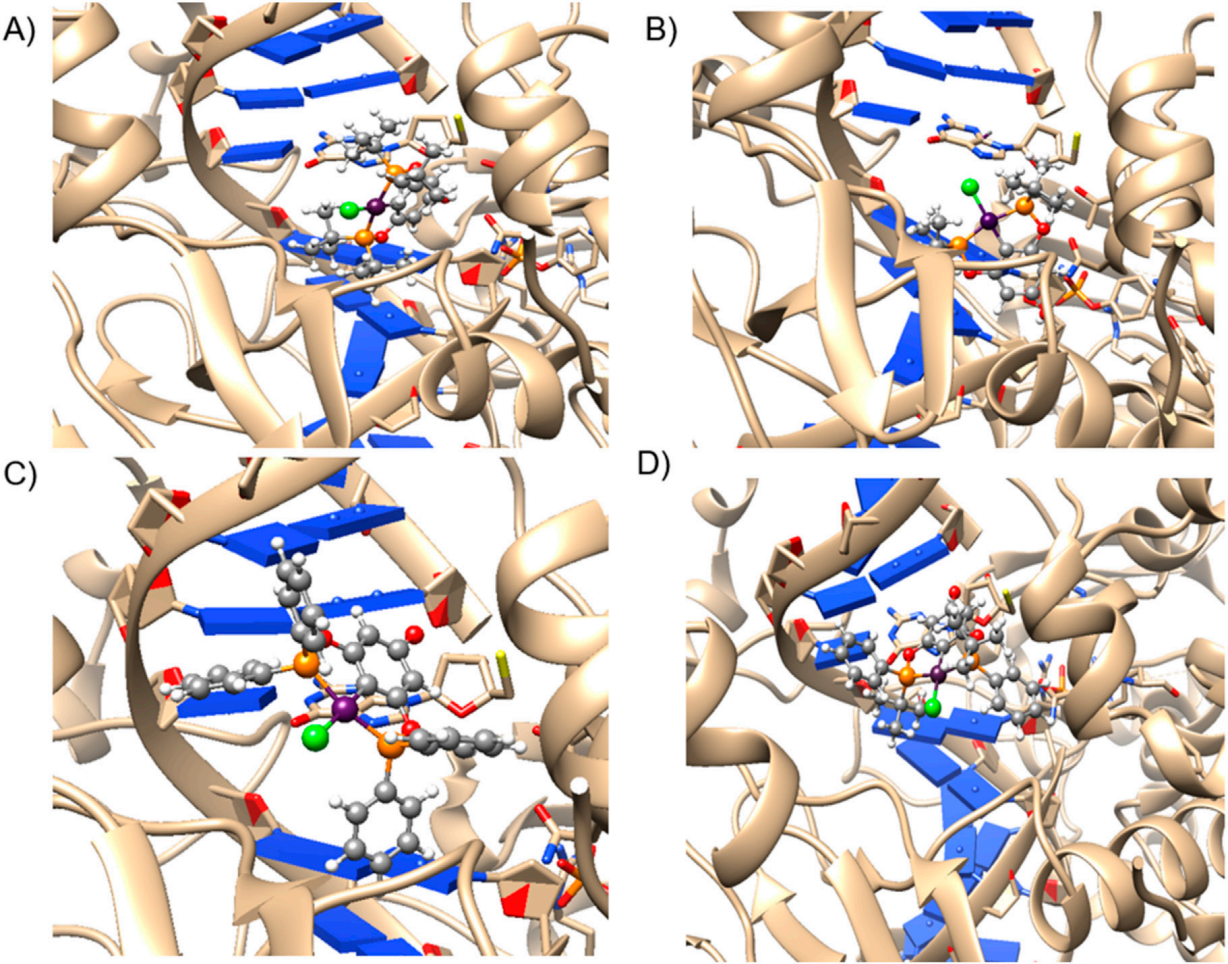
Representation of the most stable poses depicting the interaction between the Topoisomerase I model and **(A) 1a**, **(B) 1b**, **(C) 1c**, and **(D) 2** complexes.

**FIGURE 10:**
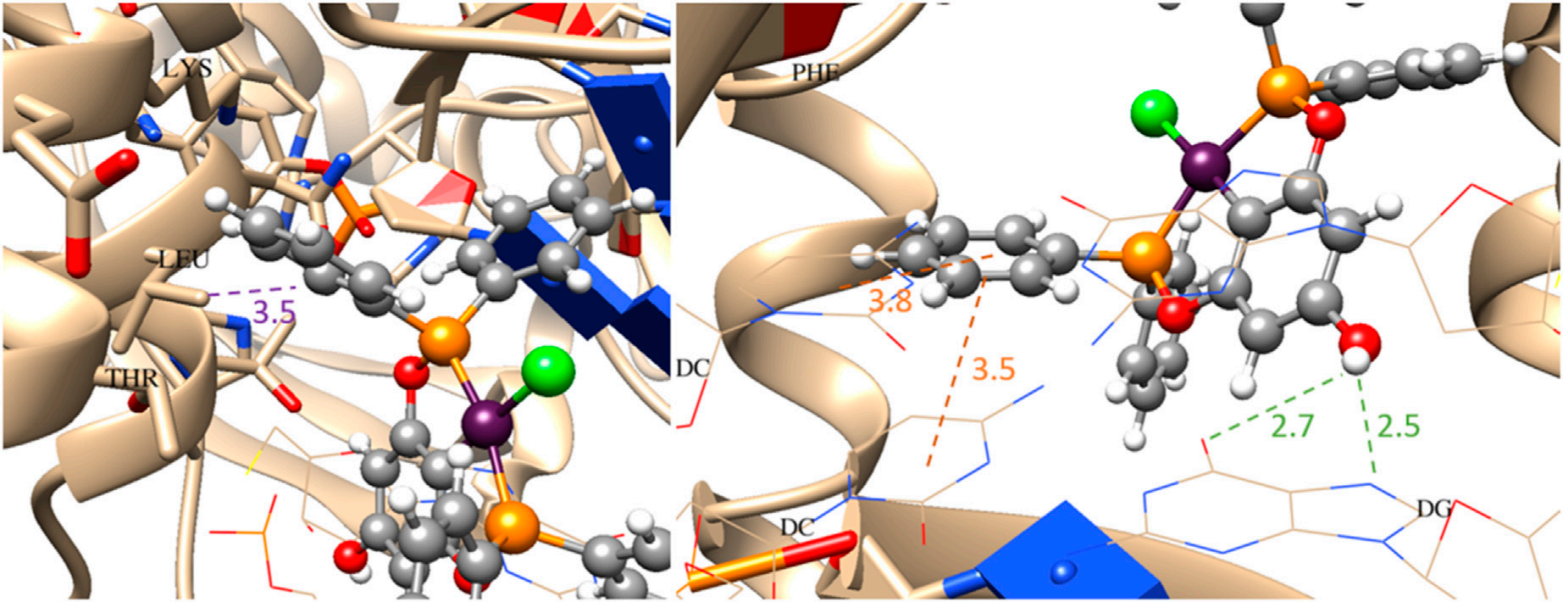
Best coordination mode of DNA model with complex **1c**. Purple π-σ interaction, green H-bond, orange π-π stacking. Distances are in Angstrom.

In the case of Topoisomerase I (Figure 9), a similar pattern of molecular docking energy to that observed with DNA is noted, displaying an Exponential Consensus Ranking (ERC) (Palacio-Rodríguez et al., 2019). comparable to that of DNA, as shown in Table 7. However, notable hydrogen bond interactions are observed between the hydroxyl group and DNA, as illustrated in Figure 10, along with hydrophobic stabilization interactions with the peptide chain of topoisomerase. This suggests that the molecular interactions contributing to the binding affinity and specificity involve not only electrostatic and π-σ interactions but also hydrogen bonding and hydrophobic interactions. These multifaceted interactions enhance the binding efficiency and specificity of the compounds towards both DNA and topoisomerase I, potentially influencing their biological activity by interfering with the normal function of these macromolecules.

**TABLE 7:** Exponential Consensus Ranking (ERC) between Topoisomerase I and the nickel complex.

Molecular docking program	1a	1b	1c	2-*rac*	Ref
Vina (kcal/mol)	−7.2	−7.4	−8.0	−8.3	−5.5
Smina (kcal/mol)	−5.8	−6.6	−8.9	−7.0	−6.5
ATD (kcal/mol)	−2.9	−2.6	−3.5	−1.0	−5.0
ERC	6.7	8.4	29.9	7.6	9.6

In the case of molecule **1b**, we observed a similar affinity behavior with the formation of identical hydrogen bonds. However, the stability of the complex is diminished due to the absence of interactions provided by aromatic groups. Conversely, with molecule **2-*rac***, there is a reduction in interactions since stabilization primarily relies on nearby aromatic rings. This underscores the critical role of aromatic rings in stabilizing these complexes. Aromatic rings significantly contribute to stabilization through π-π stacking interactions and hydrophobic effects, which are crucial for the molecular architecture of the complexes. The loss or modification of these aromatic interactions can lead to a noticeable alteration in stability and consequently affect the effectiveness of molecular interactions with the target, such as DNA or proteins like topoisomerase I.

Concerning topoisomerase II, no superior affinity was observed for the crystalline structures compared to the crystallized inhibitor obtained from the Protein Data Bank (PDB), as demonstrated in Table 8. However, like the previous targets, a superior affinity for complex **1c** was noted. This suggests that while the novel complexes did not exceed the affinity of the crystallized inhibitor for topoisomerase II, complex **1c** distinguishes itself for its consistent ability to interact more favorably across various biological targets.

**TABLE 8:** Exponential Consensus Ranking (ERC) between Topoisomerase II and the nickel complex.

Molecular docking program	1a	1b	1c	2-*rac*	Ref
Vina (kcal/mol)	−7.4	−7.1	−9.3	−7.2	−9.7
Smina (kcal/mol)	−5.3	−6.2	−9.7	−6.2	−6.5
ATD (kcal/mol)	−2.8	−2.6	−5.3	−4.6	−12.9
ERC	5.9	6.7	11.1	13.2	27.3

Complexes **1b** and **1c** exhibit superior activity against DNA structures and topoisomerase I compared to cisplatin, a well-established anticancer agent. This heightened affinity suggests that these complexes possess molecular attributes, such as π-σ interactions with phenolic rings and the capability to form hydrogen bonds, which promote stronger and more targeted interactions with these biomolecules. However, concerning topoisomerase II, the complexes did not surpass the specific inhibitor from the Protein Data Bank (PDB). This suggests that how well a compound binds and interacts closely depends on the three-dimensional structure and chemical properties of the target binding site.

The consistent affinity displayed by complex **1c** across various biological targets highlights the significance of diverse molecular interactions in docking. In addition, molecular docking studies could be correlated with the experimental cytotoxicity results in various cancer cell lines, where complex **1c** exhibited generally better activity compared to complex **1b** and cisplatin. This trend is supported by a higher binding affinity of **1c** for both DNA and topoisomerase I, with ERC values of 16.0 and 29.9, respectively. These strong interactions correlate with the higher cytotoxic efficacy of **1c** in cell lines such as U251 and K562, where it outperformed cisplatin, as well as in SK-LU-1, where it demonstrated better activity than both cisplatin and complex **1b**. Moreover, the π-σ interactions and hydrogen bonds of **1c** with DNA and topoisomerase I, identified in the molecular docking simulations, align with its higher experimental efficacy in inhibiting cancer cell replication. On the other hand, while complex **1c** consistently showed higher binding affinity for both DNA and topoisomerase I, the results with topoisomerase II revealed that it did not outperform the specific inhibitor from the PDB. This suggests that the complexes, particularly **1c**, may have a more target-specific action toward DNA and topoisomerase I, rather than being broad-spectrum inhibitors. The reduced affinity of the complexes for topoisomerase II compared to DNA and topoisomerase I highlights the importance of target-specific interactions in determining biological activity, explaining why complex **1c** does not universally outperform in all assays but shows selectivity depending on the target.

## 7 Conclusion

In this study, four POCOP-Ni(II) pincer compounds (**1a**, **1b**, **1c**, and **2**) were evaluated as potential agents with anticancer and antioxidant properties. A novel structure (Complex **2**), synthesized from phloroglucinol and the corresponding phosphine, and its racemic isomer **2-*rac*** was meticulously characterized using single-crystal X-ray diffraction (DRX), revealing a distinctive distorted square-planar geometry. The supramolecular interactions identified in the crystalline packing underscore the significance of H•••Cl, O•••H interactions, and van der Waals H•••H contacts in the structure. In fact, mainly the OH•••Cl interactions play a critical role in the four structures to stabilize polymeric interactions and favor crystallization.

Cytotoxic evaluation demonstrated that complexes **1b** and **1c** exhibited significant antiproliferative activity against a spectrum of cancer cell lines (U251, K562, HCT-15, MCF-7, and SK-LU-1), with IC_50_ values ranging from 2.43 to 7.85 μM. In contrast, complex **1a** showed the highest antioxidant activity, with an IC_50_ value of 1.55 μM. Furthermore, a competitive fluorescent displacement assay revealed that complexes **1a-c** could effectively displace EB from the DNA-EB adduct.

Molecular docking studies of complexes **1b** and **1c** suggests that these compounds could be effective anticancer treatments, demonstrating strong interactions with DNA and topoisomerase I compared to standard treatments such as cisplatin. However, their performance against topoisomerase II, relative to specific inhibitors from the Protein Data Bank, underscores the complexity of drug development and the necessity for a comprehensive understanding of drug-target interactions. Complex **1c** consistently exhibited strong affinity across various targets, indicating its potential for diverse therapeutic applications. These findings emphasize the importance of employing a multidisciplinary approach to advance these complexes into effective anticancer therapies, addressing a critical need in cancer treatment.

These findings underscore the multifaceted potential of the POCOP-Ni(II) pincer compounds studied, with distinct complexes demonstrating promising anticancer and antioxidant properties. The elucidation of their structural characteristics and interactions offers valuable insights into their potential applications in therapeutic and biomedical contexts. Continued exploration will be crucial for unraveling the complex mechanisms and applications of these compounds in medical chemistry and therapeutic development.

## 8 Experimental section

All chemical compounds were commercially obtained and used as received without further purification. The ^1^H, ^13^C{^1^H}, ^31^P{^1^H} NMR spectra were obtained on a Bruker Avance III 400 MHz spectrometer and a Bruker 500 Ascend spectrometer. Chemical shifts are reported in ppm down field of TMS employing the residual signals in the solvent (CDCl_3_) as internal standard. ATR-IR measurements were performed on a FTIR NICOLET IS50, Thermo Fisher Scientific Spectrometer. Elemental analyses were made on a Thermo Scientific Flash 2000 elemental analyzer, using a Mettler Toledo XP6 Automated-S Microbalance and sulfanilamide as standard (Thermo Scientific BN 217826, attained values N = 16.40%, C = 41.91%, H = 4.65%, and S = 18.63%; certified values N = 16.26%, C = 41.81%, H = 4.71%, and S = 18.62%). MS-DART determinations were recorded in a JEOL The AccuTOF JMS-T100LC Mass spectrometer. Complexes **1a, 1b,** and **1c** were synthesized according to the methodology previously designed in our investigation group.^21^ They were characterized by ^1^H, ^13^C, and ^31^P NMR spectroscopy, and elemental analysis showing the expected results.

### 8.1 Synthesis of compounds 1a-c

The pincers compounds **1a-c** (R = ^*t*^Bu, ^*i*^Pr, Ph, respectively) were synthesized according to the methodology reported by our investigation group (García-Eleno et al., 2015). And spectroscopic analysis was entirely correlated.

### 8.2 Synthesis of compound 2

A Schlenk flask was charged with 1 mmol of phloroglucinol, 2 mmol of chloro (tert-butyl)phenylphosphine, 2 mmol NEt_3_, and dry THF (30 mL); under a nitrogen atmosphere. Then, the solution was refluxed overnight and evaporated under a vacuum. The crude product was purified by chromatographic column using CH_2_Cl_2_ as eluent. The compound was obtained as a yellow solid. Yield 97%. M. p. 143°C–144 °C. ^1^H NMR (500 MHz, CDCl_3_) δ 8.13–8.06 (m, 4H, C*H*Ar), 7.50–7.43 (m, 6H, C*H*Ar), 6.11 (s, 1H, C*H*Ar), 6.11 (s, 1H, C*H*Ar), 1.37–1.27 (m, 18H, -C(C*H*_3_)_3_). ^13^C{^1^H} NMR (125.7 MHz, CDCl_3_) δ 167.9 (m, *C*-O), 167.7 (m, *C*-O), 157.6 (s, C-O), 137.8 (s, *C-*H), 131.7 (s, *C*-H), 131.2 (s, *C*-H), 131.1 (s, *C*-H), 130.9 (m, *C*-P), 130.7 (m, *C*-P), 128.3 (s, *C*-H), 128.2 (s, *C*-H), 94.5 (t, *C*-H, ^*3*^ *J*_*C-P*_ = 6.5 Hz), 113.7 (m, *C*-Ni), 94.4 (t, *C*-H, ^*3*^ *J*_*C-P*_ = 6.5 Hz), 37.5 (t, -*C*(CH_3_)_3_, ^*1*^ *J*_*C-P*_ = 12.2 Hz), 37.0 (t, -*C*(CH_3_)_3_, ^*1*^ *J*_*C-P*_ = 12.7 Hz), 25.5 (s, -CH(*C*H_3_)_2_), 25.4 (s, -CH(*C*H_3_)_2_). ^31^P{^1^H} NMR (202.4 MHz, CDCl_3_): δ 160.56, 159.71. MS (DART): m/z 547 [M + H]^+^. IR (ATR, cm^−1^): 3371 (b, -OH), 1130 (s, C-O-C). Elem. Anal. Calcd. for C_26_H_31_ClNiO_3_P_2_: C, 57.03; H, 5.71. Found: C, 56.26; H, 6.93.

### 8.3 Cytotoxic evaluation

The compounds were screened *in vitro* against human cancer cell lines: HCT-15 (human colorectal adenocarcinoma), MCF-7 (human mammary adenocarcinoma), K562 (human chronic myelogenous leukaemia), U251 (human glioblastoma), PC-3 (human prostatic adenocarcinoma), SK-LU-1 (human lung adenocarcinoma), COS-7 (cell line monkey African green kidney) cell lines were supplied by the National Cancer Institute (United States of America) and were donated by the Cancer Institute of Mexico. The cell lines were cultured in RPMI-1640 medium supplemented with 10% fetal bovine serum, 2 mM L-glutamine, 25 μg/mL amphotericin B (Gibco) and 1% non-essential amino acids (Gibco). They were maintained at 37 °C in a humidified atmosphere with 5% CO2.

Cytotoxicity after treatment of the tumors cells and normal cells with the test compounds was determined using the protein-binding dye sulforhodamine B (SRB) in a microculture assay to measure cell growth (Vichai and Kirtikara, 2006). The cultures were exposed for 48 h to the compound at concentrations 25 μM. After the incubation period, cells were fixed to the plastic substratum by addition of 50 μL of cold 50% aqueous trichloroacetic acid. The plates were incubated at 4°C for 1 h, washed with tap H2O, and air-dried. The trichloroacetic-acid-fixed cells were stained by the addition of 0.4% SRB. Free SRB solution was the removed by washing with 1% aqueous acetic acid. The plates were then air-dried, and the bound dye was solubilized by the addition of 10 mM unbuffered tris base (100 μL). The plates were placed on and shaken for 10 min, and the absorption was determined at 515 nm using an ELISA plate reader (Bio-Tex Instruments). The inhibitory concentration 50 (IC_50_) values were calculated on extrapolated fit curves based on doses/response data analysed for each compound through lineal regression analysis.

### 8.4 Lipid peroxidation inhibition

#### 8.4.1 Animals

Adult male Wistar rat (200–250 g) was proveed by Instituto de Fisiología Celular, Universidad Nacional Autónoma de México (UNAM). Procedures and care of animals were conducted in conformity with Mexican Official Norm for Animal Care and Handling (NOM-062-ZOO-1999). They were maintained at 23°C ± 2°C on a 12/12 h light-dark cycle with free access to food and water.

#### 8.4.2 Rat brain homogenate preparation

Animal euthanasia was carried out avoiding unnecessary pain with CO_2_. The cerebral tissue (whole brain) was rapidly dissected and homogenized in phosphate buffered saline (PBS) solution (0.2 g of KCl, 0.2 g of KH_2_PO_4_, 8 g of NaCl, and 2.16 g of NaHPO_4_ ·7 H_2_O/l, pH adjusted to 7.4) as reported elsewhere (Rossato et al., 2002; Domínguez et al., 2005) to produce a 1/10 (w/v) homogenate. Homogenate was centrifuged for 10 min at 800 rcf (relative centrifugal field). The supernatant protein content was measured using the Folin and Ciocalteu’s phenol reagent (Lowry et al., 1951) and adjusted wih PBS at 2.66 mg of protein/mL.

As an index of lipid peroxidation, TBARS levels were measured using rat brain homogenates according to the method described by Ng et al. (2000), with some modifications. Supernatant (375 µL) was added with 50 µL of 20 µM EDTA and 25 µL of each sample concentration solved in DMSO (25 µL of DMSO for control group) and incubated at 37°C for 30 min. Lipid peroxidation was started adding 50 µL of freshly solution FeSO_4_ 100 µM and incubated at 37°C for 1 h. The TBARS content was determined as described by Ohkawa et al. (1979) with 500 µL of TBA reagent (0.5% 2-thiobarbituric acid in 0.05 N NaOH and 30% trichloroacetic acid, in 1:1 proportion) was added at each tube and cooled on ice for 10 min, centrifugated at 13,400 rcf for 5 min and heated at 80°C in a water bath for 30 min. After cooling at room temperature, the absorbance of 200 µL of supernatant was measured at ⌊ = 540 nm in a Bio-Tek Microplate Reader Synergy HT. Concentration of TBARS was calculated by interpolation in a standard curve of tetra-methoxypropane (TMP) as a precursor of MDA (Esterbauer and Cheeseman, 1990). Results were expressed as nmoles of TBARS per mg of protein. The inhibition ratio (I_R_ [%]) was calculated using the following formula *I*_*R*_ =(C-E)*100/C, where *C* is the absorbance of control and *E* is the absorbance of the test sample. Butylated hydroxytoluene (BHT) and α-tocopherol were used as positive standards.

All data were represented as mean ± standard error (SEM). Data were analyzed by one-way ANOVA followed by Dunnett´s test for comparison against control. Values of *p* ≤ 0.05 (*) and *p* ≤ 0.01 (**) were considered statistically significant. The inhibitory concentration 50 (IC_50_), was estimated by means of a linear regression.

### 8.5 Competitive displacement assay

A 4 mM working solution of salmon sperm DNA (ss-DNA) (SIGMA) was prepared in 5 mM Tris-HCl and 5 mM NaCl buffer at pH 7.4 (Backman-Blanco et al., 2020) Compounds **1a**, **1b** and **1c** were dissolved in DMSO at concentrations of 10, 6.66 and 3.3 mM respectively. To get insight whether compounds **1a**, **1b** and **1c** may interact with DNA, an ethidium bromide (EB) displacement assay was performed as mentioned in the literature (Banerjee et al., 2013). Briefly, a 3 mL buffer containing 5 mM Tris-HCl, 5 mM NaCl buffer at pH 7.4 and 5.0 × 10^−5^ M EB was mixed in a 1 cm fluorescence cuvette with 2.5 × 10^−4^ M of ss-DNA. The cuvette was placed in an Agilent Cary Eclipse spectrofluorometer and titrated with different amounts of the stock solution of the compounds **1a**, **1b** and **1c**, after thorough mixing the fluorescence spectra were recorded at 25 °C in the range of 540 and 700 nm (⌊_ex_ = 520 nm).

### 8.6 Computational details for compounds 1a, 1b, 1c and 2-*rac*

Electronic structure calculations were carried out at the B3LYP/6-31+G (d,p) level of theory. Initial ligand geometries were extracted from crystal structures, subsequently optimized, and subjected to frequency calculations to validate their status as minima on the potential energy surface. To account for solvent effects (water), the SMD continuum method was employed. Atomic charges, essential for molecular docking simulations, were obtained using the NPA scheme (Reed et al., 1985). All the electronic structure calculations were carried out using the Gaussian16 suite of programs (Frisch et al., 2016).

To optimize docking outcomes, minimize the influence of force fields, and reduce system dependency, we employed an Exponential Consensus Ranking (ECR) (Palacio-Rodríguez et al., 2019). This method computes a consensus score, P(i), for each molecule by aggregating exponential ranks from various programs. This consensus approach was applied using three molecular docking programs: AutoDock 4, (Morris et al., 2009), AutoDock Vina, (Trott and Olson, 2010), and Smina, (Koes et al., 2013), which incorporates the Vinardo scoring function.

The molecular docking simulation was performed utilizing DNA models, as well as Topoisomerase I and II, which are crucial targets for contemporary cancer treatments. The three-dimensional structures of DNA models and Topoisomerases I and II were retrieved from PDB codes 1AIO, (Takahara et al., 1995), 1T8I, (L. Staker et al., 2005) and 5GWK, (Wang et al., 2017), respectively. These structures have been previously utilized in computational cancer research. (Backman-Blanco et al., 2020; Jamal, 2020; Madeddu et al., 2022). Receptor file preparation was conducted using AutoDock Tools 1.4.5, (Morris et al., 2009), involving the removal of water molecules, the addition of all hydrogen atoms with nonpolar hydrogens merged into carbon atoms, and incorporation of Gasteiger charges into the receptor models, (Gasteiger and Marsili, 1980), resulting in pdbqt files. Ligand charges were determined from NPA population analysis based on density functional theory calculations. Docking experiments utilized a grid box measuring 60 × 60 × 60 Å³ along the X, Y, and *Z*-axes with a spacing of 0.375 Å. Analysis of ligand-receptor complexes was performed using Chimera, (Pettersen et al., 2004), PyMol, (Schrödinger, 2015), and Maestro Schrodinger (Schrödinger Release, 2023-2, 2023) programs.

### 8.7 Data collection and refinement for compound 2-*rac*

All crystals were grown by slow evaporation of CH_2_Cl_2_, then placed on a Bruker Smart Apex II diffractometer with a Mo-target X-Ray source (λ = 0.71073 Å). The detector was placed at 5.0 cm from the crystals and frames were collected with a scan width of 0.5 cm in ω and an exposure time of 10 s/frame. Frames were integrated with the Bruker SAINT software package using a narrow-frame integration algorithm. Non-systematic absences and intensity statistics were used for space group determination of orthorhombic unit cell for **2-*rac***. The structures were solved using Patterson methods using the SHELXS-2014/7 program (Bruker AXS Inc, 2018). The remaining atoms were located *via* a few cycles of least squares refinements and difference Fourier maps. Hydrogen atoms were input at calculated positions and allowed to ride on the atoms to which they were attached. Thermal parameters were refined for hydrogen atoms on the phenyl groups using a Ueq = 1.2 Å to precedent atom. The final cycles of refinement were carried out on all non-zero data using SHELXL-2014/7 (Sheldrick, 2015). Absorption corrections were applied using the SADABS program (Krause et al., 2015).

## Data Availability

The original contributions presented in the study are included in the article/Supplementary Material; further inquiries can be directed to the corresponding author.
