# Supplementary material for: POCOP-Ni(II) pincer compounds derived from phloroglucinol. Cytotoxic and antioxidant evaluation
Source: Front Chem. 2024 Nov 20;12:1483999. doi: 10.3389/fchem.2024.1483999 (PMC11614598; doi:10.3389/fchem.2024.1483999)
Supplement: Supplementary file 1 [file DataSheet1.docx]

*Supporting Information*

**POCOP-Ni(II) Pincer Compounds Derived from Phloroglucinol. Cytotoxic and Antioxidant Evaluation**

**Table of contents**

1. Crystal data and details of the structure determination of compounds **2-*rac***…….. 2
2. ^1^H NMR spectrum of **2** (500 MHz, CDCl_3_)……………………………………… 3
3. ^13^C{^1^H} NMR spectrum of **2** (125.7 MHz, CDCl_3_)……………………………… 3
4. ^31^P{^1^H} NMR spectrum of **2** (202.4 MHz, CDCl_3_)……………………………… 4
5. ATR-FTIR spectrum of **2**………………………………………………………… 4
6. MS (DART) of compound **2**……………………………………………………… 5
7. 1D fragment of compounds **1a, 1b, 1c** and **2-*rac*** formed by OH···Cl interaction [the set graph of four compounds are equal to C(8)]…………………………….. 5
8. Inhibition of rat brain lipid peroxidation…………………………………………………. 6
9. Root Mean Square Deviation (RMSD) in Å between crystallographic and optimized structures using the B3LYP/6-31+G(d,p) method. Blue denotes the crystal structure, while brown indicates the optimized structure………………………………………………… 7
10. **X-ray crystallography**

**Table S1.** Crystal data and details of the structure determination of compounds **2-*rac*.**

| Compound | **2-*rac*** |
| --- | --- |
| Empirical formula | C_26_H_31_ClNiO_3_P_2_ |
| Formula weight | 547.61 |
| Temperature (K) | 298(2) |
| Wavelength (Å) | 0.71073 |
| Crystal system | Orthorhombic |
| Space group | Pbca |
| Unit cell dimensions | a = 19.3498(12) Å  b = 13.8767(14) Å  c = 19.6290(13) Å  α = 90 °  β = 90 °  γ = 90 ° |
| Volume (Å^3^) | 5269.8(7) |
| Z | 8 |
| Density (calc.) (Mg/m^3^) | 1.380 |
| Absorption coefficient (mm^-1^) | 0.984 |
| F(0 0 0) | 2288 |
| Crystal size (mm^3^) | 0.350 x 0.240 x 0.070 |
| Theta range for data collection (º) | 3.596 to 29.560 ° |
| Index ranges | -24<=h<=24,  -11<=k<=17,  -18<=l<=27 |
| Reflections collected | 18891 |
| Independent reflections | 6410 [R(int)= 0.0456] |
| Completeness to theta = 25.242º | 99.7 % |
| Absorption correction | Semi-empirical from equivalents |
| Refinement method | Full-matrix least-squares on F^2^ |
| Data/restraints/parameters | 6410 / 0 / 304 |
| Goodness-of-fit on F^2^ | 1.010 |
| Final *R* indices [I > 2sigma(I)] | R1 = 0.0500, wR2 = 0.0996 |
| *R* indices (all data) | R1 = 0.1037, wR2 = 0.1262 |
| Largest diff. peak and hole (e.Å^-3^) | 0.917 and -0.637 |

**2. Spectroscopic Data**

**Figure S1**. ^1^H NMR spectrum of **2** (500 MHz, CDCl_3_).

**Figure S2**. ^13^C{^1^H} NMR spectrum of **2** (125.7 MHz, CDCl_3_).

**Figure S3**. ^31^P{^1^H} NMR spectrum of **2** (202.4 MHz, CDCl_3_).


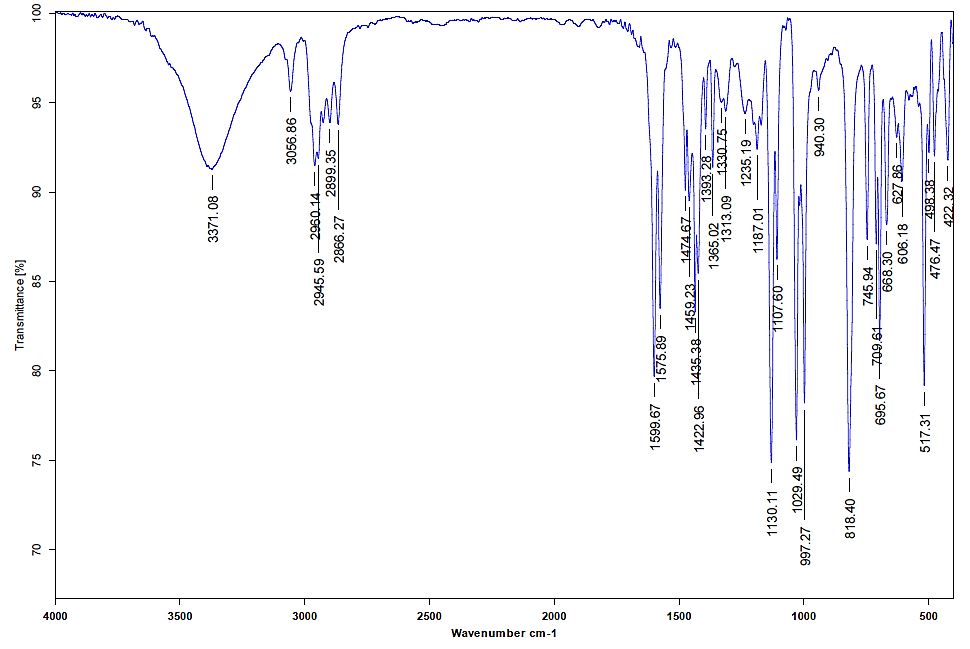


**Figure S4**. ATR-FTIR spectrum of **2**.

**
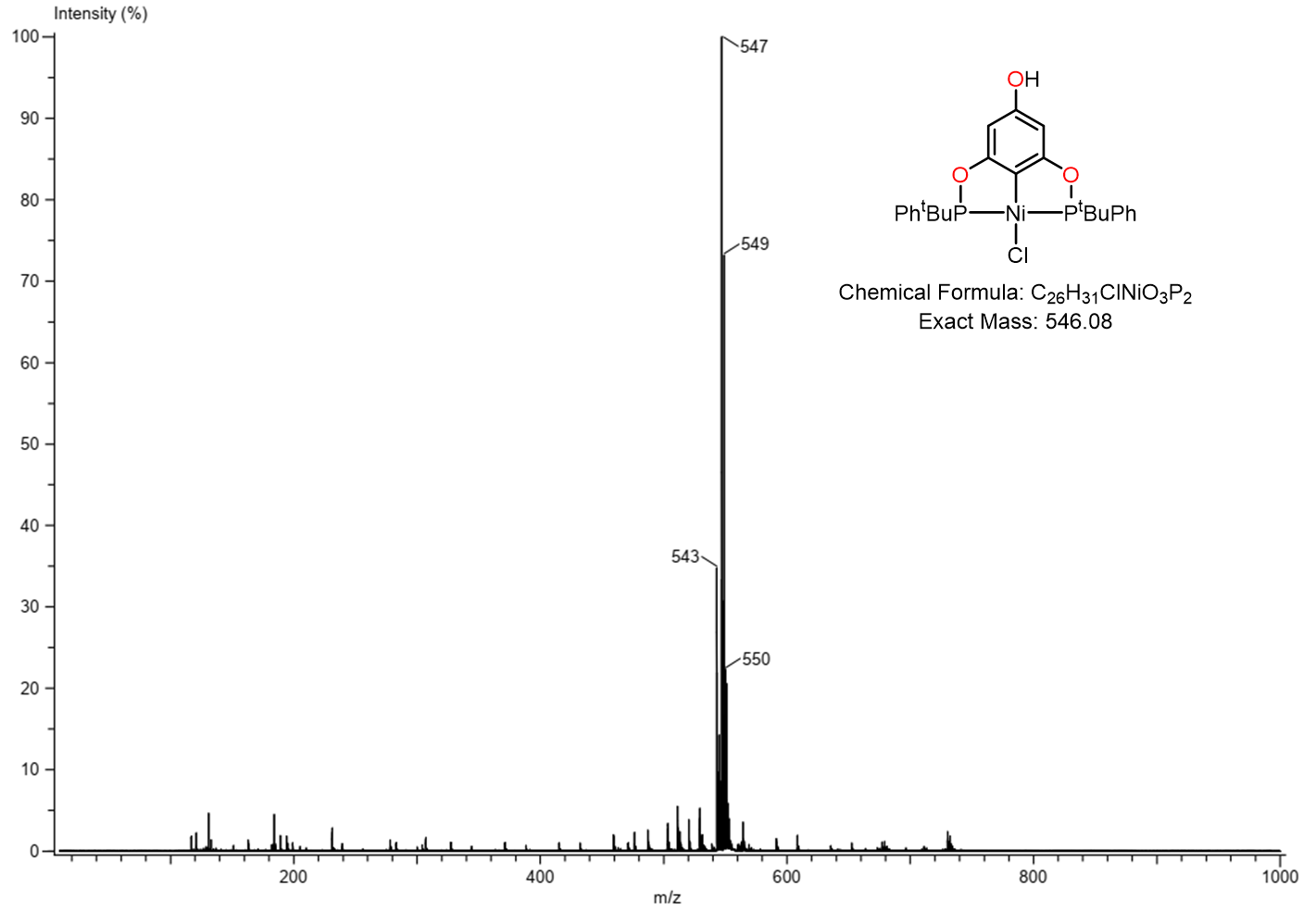
**

**Figure S5**. MS (DART) of compound **2**.


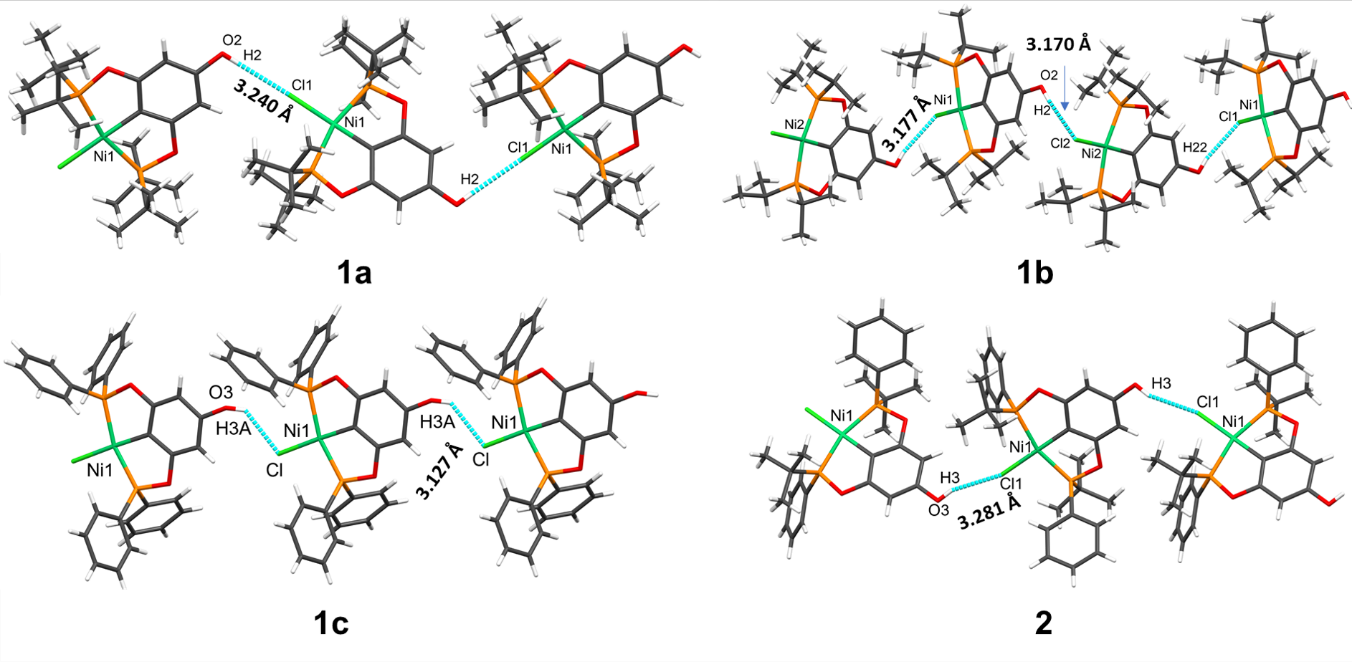


**2-rac**

**Figure S6.** 1D fragment of compounds **1a, 1b, 1c** and **2-*rac*** formed by OH···Cl interaction [the set graph of four compounds are equal to C(8)].

1. **Biological assays**

**Table S2**. Inhibition of rat brain lipid peroxidation

| **Sample** | **Concentration**  **(µM)** | **TBARS**  **(nmol/mg)** | **Inhibition**  **(%)** | **IC_50_** |
| --- | --- | --- | --- | --- |
| **1a**  (n=3) | Basal  Control  1  1.33  1.78  2.37  3.16 | 0.394+-0.135  9.844±0.740  7.446±0.744  6.206±0.804*  3.117±1.132**  0.684±0.085**  0.463±0.024** | 24.63±1.82  37.42±3.43*  69.5±8.85**  93.1±0.36**  95.29±0.13** | 1.55±0.08 |
| **1b**  (n=3) | Basal  Control  1  3.16  10  31.62  100 | 0.677±0.217  9.968±0.380  9.636±0.492  8.306±0.318*  6.479±0.580**  0.751±0.144**  0.462±0.075** | 3.42±1.45  16.66±1.03*  35.24±3.74**  92.55±1.21**  95.39±0.64** | 13.30±0.77 |
| **1c**  (n=3) | Basal  Control  1  3.16  10  31.62  100 | 0.677±0.217  9.968±0.380  9.438±0.429  8.740±0.430  6.961±0.538**  2.728±1.016**  0.705±0.131** | 5.36±0.73  12.36±1.57  30.36±3.14**  73.12±9.74**  92.98±1.16** | 19.29±3.04 |
| **2** | Basal  Control  0.56  1  1.78  3.16  5.62  10 | 0.299±0.080  10.940±0.586  9.755±0.207*  9.184±0.176**  7.598±0.346**  1.249±0.076**  0.414±0.013**  0.374±0.029** | 10.55±3.01*  15.76±3.16**  30.40±2.82**  88.51±0.98**  96.18±0.33**  96.53±0.46** | 2.19±0.05 |
| **BHT**  (n=5) | Basal  Control  0.56  0.75  1  1.33  1.78  2.37 | 0.268±0.053  7.384±0.630  6.098±0.353  5.559±0.294*  4.457±0.283**  3.228±0.572**  1.315±0.489**  0.487±0.075** | 16.64±2.86  23.92±2.69*  37.14±7.44**  53.59±8.93**  81.59±6.89**  93.16±1.16** | 1.22±0.44 |
| **α-Tocopherol**  (n= 4) | Basal  Control  0.32  1  3.16  10  31.62  100 | 0.200±0.011  6.589±0.213  6.048±0.242  5.211±0.332*  3.676±0.569**  2.725±0.335**  1.849±0.319**  1.408±0.364** | 8.26±1.31  21.13±2.56*  44.84±6.74**  59.00±3.71**  72.30±3.87**  79.09±4.79** | 6.78±2.16 |

Homogenised in: PBS; Vehicle: DMSO; Experiment: Curve; Peroxidation: induced with FeSO_4_ 10 µM, Incubation time: 1 h; EDTA: 2 µM.

The values represent the average of three independent experiments ± standard error of the media$\left(\overline{x}\pm ES\right)$. Data were subjected to an analysis of variance (ANOVA) followed by a Dunnett pass to isolate groups with significant differences. The values of *p* ≤ 0.05 (*) and *p* ≤ 0.01 (**) were considered as significant differences with respect to control.

**Table S3.** Root Mean Square Deviation (RMSD) in Å between crystallographic and optimized structures using the B3LYP/6-31+G(d,p) method. Blue denotes the crystal structure, while brown indicates the optimized structure.

| Ligand | 1a | 1b | 1c | 2-*rac* |
| --- | --- | --- | --- | --- |
| RMSD (Å) | 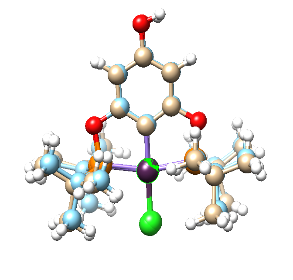0.12 | 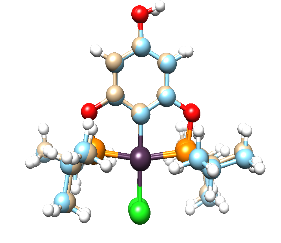  0.15 | 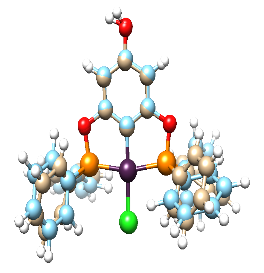0.25 | 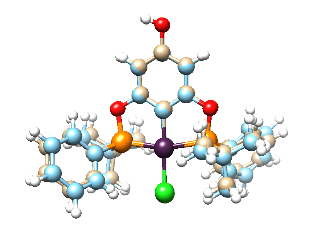  0.23 |
